# From Cyclo[18]carbon to the Novel Nanostructures—Theoretical Predictions

**DOI:** 10.3390/ijms232112960

**Published:** 2022-10-26

**Authors:** Agnieszka Brzyska, Tomasz Panczyk, Krzysztof Wolinski

**Affiliations:** 1 Jerzy Haber Institute of Catalysis and Surface Chemistry, Polish Academy of Sciences, Niezapominajek 8, 30-239 Krakow, Poland; 2 Institute of Chemical Sciences, Faculty of Chemistry, Maria Curie Sklodowska University in Lublin, pl. Maria Curie-Sklodowska 3, 20-031 Lublin, Poland

**Keywords:** cyclo[18]carbon, carbonaceous materials, low HOMO–LUMO gap structures, novel nanostructures

## Abstract

In this paper, we present a number of novel pure-carbon structures generated from cyclo[18]carbon. Due to the very high reactivity of cyclo[18]carbon, it is possible to link these molecules together to form bigger molecular systems. In our studies, we generated new structures containing 18, 36 and 72 carbon atoms. They are of different shapes including ribbons, sheets and tubes. All these new structures were obtained in virtual reactions driven by external forces. For every reaction, the energy requirement was evaluated exactly when the corresponding transition state was found or it was estimated through our new approach. A small HOMO–LUMO gap in these nanostructures indicates easy excitations and the multiple bonds network indicates their high reactivity. Both of these factors suggest that some potential applications of the new nanostructures are as components of therapeutically active carbon quantum dots, terminal fragments of graphene or carbon nanotubes obtained after fracture or growing in situ in catalytic reactions leading to the formation of carbonaceous materials.

## 1. Introduction

Some evidences of the existence of the C_18_ compound in the gas phase have been reported since 1992 [1,2,3] but it was synthesized and isolated for the first time in 2019 by Kaiser et al. [4]. Before that, this molecule has been the subject of numerous theoretical studies on its geometric and electronic structure [5,6,7]. Depending on the level of theory, two types of electronic structures have been predicted: polyynic with alternating single and triple bonds and cumulenic with double bonds [8,9,10,11,12,13,14] (see Figure S1 in SI [15]). The cumulenic structure of C**_18_** is found at the popular DFT/B3LYP level of theory and also at the Moller–Plesset (MP2) level [5,9,10,12,15,16,17,18,19], while the Hartree–Fock (HF) and Coupled Cluster (CC) methods as well as DFT potentials with larger amounts of the exact HF exchange potential, lead to the polyynic form [7,20,21,22]. The conclusion from the latest experimental work based on high-resolution atomic force microscopy [2] is that the cyclo[18]carbon structure is polyynic rather than cumulenic. Very recently, new theoretical papers dealing with various aspects of the cyclo[18]carbon molecule have been published. They described its ground and excited state electronic structures, its very high reactivity as well as its very unique mechanical properties [20,23].

Due to the high reactivity of cyclo[18]carbon, one can expect that these molecules should be easily linked into larger systems and form nanostructures with potential biomedical applications such as quantum carbon dots [24,25]. The authors in ref. [4] have made a statement that their discovery of cyclo[18]carbon opens *an avenue for the synthesis of other carbon allotropes and carbon-rich materials from the coalescence of cyclocarbon molecules*. We went along that avenue in a theoretical manner. Starting from cyclo[18]carbon, we generated a number of novel structures. These new structures containing up to 72 carbon atoms include ribbons, sheets and tubes. All the new structures were obtained via the virtual chemical reactions driven by external forces.

In these studies, we used external forces in the framework of the enforced geometry optimization (EGO) method [26] to induce specific chemical reactions. The basic idea of the EGO method is to perform geometry optimization in the presence of external forces acting on selected nuclei in a molecule. In general, external forces can be applied to an arbitrary number of atoms in arbitrary directions. In practice, these forces are usually applied to a pair (or pairs) of atoms and act along the line joining these two atoms in a “push” or “pull” mode. These forces are constant in magnitude, but their directions are changing according to the atom’s positions during geometry optimization. The EGO method was successfully applied in many cases including conformational transitions in the pyranose ring [27], isomerization of stilbene and azobenzene [28,29] and also in studies of structural changes due to mechanical stress in polysaccharides [30,31,32,33,34]. In the current study, we apply that methodology for initiating chemical reactions within or between cyclo[18]carbons and analyze the chemical properties of several new carbon allotropes obtained in such a way.

Carbon-only structures with different ring topologies may exist and this can be verified experimentally, for example, a biphenylene network composed of four-, six- and eight-membered carbon rings [35,36]. Recently, complex carbon nanostructures have been found in diamond [37]. Another sheet structure is γ-graphyne, which is composed of benzene rings joined hexagonally by carbon–carbon triple bonds from each carbon atom [38].

In these studies, the new structures were generated from the cyclo[18]carbon. This molecule has been recently synthesized using very sophisticated techniques involving single-atom manipulations within the atomic force microscopy (AFM) framework [39,40]. In our theoretical studies, we also used a kind of AFM approach by applying external forces to selected atoms in a molecular system. The EGO method was used to run virtual reactions where two smaller subsystems were linked together to form a bigger system. This way, starting from C18, we generated a series of C36 and C72 novel structures of different types including ribbons, sheets and tubes. All reported structures corresponded to the stationary points on potential energy surfaces (PESs) which was verified by vibrational analysis.

We begun the present project in two ways. In the first one, we linked together two cyclo[18]carbon molecules and then connected the resulting products in various manners. The first two C_18_ were linked by pushing together two pairs of neighboring carbons, each pair from one of the two cyclo[18]carbon molecules. As a result, two C18 molecules were connected with two bonds.

In the second way, one cyclo[18]carbon molecule was first squeezed by pushing together two single carbons or two pairs of carbons from opposite sides of the C_18_ circle. This way, one or two C–C bonds were formed inside the C_18_ circle. Following this, we built up bigger structures by bonding together these squeezed C_18_ molecules.

**Figure 1:**
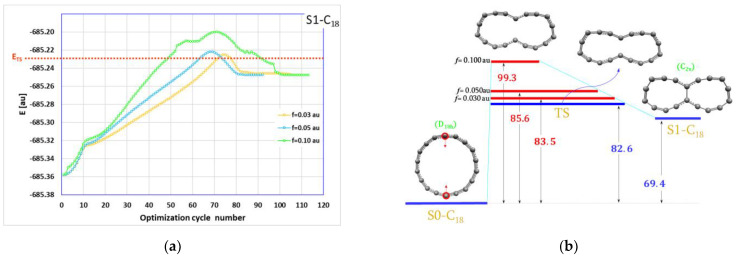
(**a**) The algorithmic reaction path (the geometry optimization history) with the marked transition state energy value; (**b**) the energy diagram with the estimated upper limit of the energy demand for the formation of the S1-C_18_ molecule. All values in the energy diagrams are in kcal/mol.

**Figure 2:**
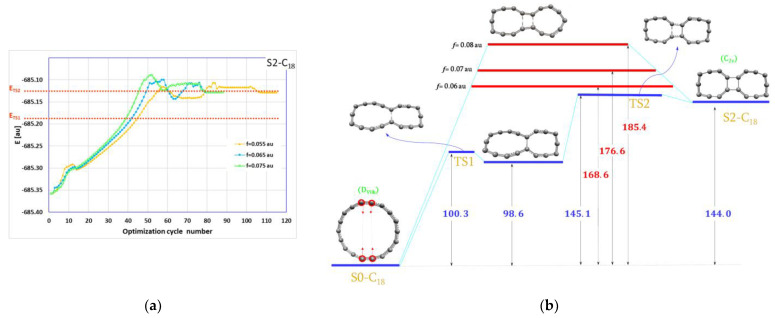
(**a**) The algorithmic reaction path (the geometry optimization history) with the marked transition state energy values; (**b**) the energy diagram with the estimated upper limit of the energy demand for the formation of the S2-C_18_ molecule. All values in the energy diagrams are in kcal/mol.

Thus, we have three initial C_18_ structures: squeezed with two bonds inside, squeezed with one bond and unsqueezed cyclo[18]carbon. We denote these structures with the S2, S1 and S0 symbols, respectively: S2-C_18_, S1-C_18_ and S0-C_18_. These structures are shown in Figure 1 and Figure 2.

Note that by squeezing C_18_, new moieties are introduced: 9-membered and 4-membered rings in S2-C_18_ and 10-membered rings in S1-C_18_. The next step in our studies was to link together two such initial structures to form S0-C_36_, S1-C_36_ and S2-C_36_. In the last step, we doubled the C_36_ units and generated the S0-C_72_, S1-C_72_ and S2-C_72_ structures. It should be emphasized that two C_18_ as well as two C_36_ can be pushed together in various orientations which may lead to the structures of different shapes: C_36-ribbon_, C_36-sheet_, C_72-ribbon_ and C_72-sheet_ (we did not consider layers). Some of the C_72-sheet_ structures were also rolled up to form tubes C_72-tube_.

## 2. Results and Discussion

### 2.1. Polyynic versus Cumulenic

We begun with checking how the polyynic and cumulenic forms of cyclo[18]carbon (from hereafter called also C**_18_**) influence the electronic structure of C_36_ when two C**_18_**are linked together. It is quite obvious that the cumulenic structure of C**_18_**must be destroyed when perturbed by any new bond. Nevertheless, we examined this issue by additional calculations.

We have constructed a user-defined DFT/USER exchange-correlation potential with 0.55 of exact HF exchange, 0.45 of Slater local exchange and 1.0 of the Lee–Yang–Parr’s non-local correlation. With DFT/USER and DFT/B3LYP, geometry optimization of C**_18_**led to the polyynic (C**_18-p_**) and cumulenic (C**_18-c_**) forms with C–C bonds of 1.336/1.221 and 1.285 Å, respectively. Then, using the EGO method, two C**_18_** were pushed together with external forces acting on two pairs of C–C atoms, each pair from another C**_18_**unit:

C_18-p_→→→^pushing forces^→→→C_18-p_→→C_36-p_ EGO with DFT/USER

C_18-c_→→→^pushing forces^→→→C_18-c_→→C_36-c_ EGO with DFT/B3LYP

**Figure 3:**
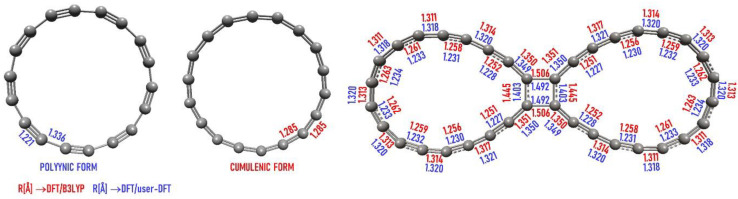
The bond lengths for the polyynic and cumulenic forms of cyclo[18]carbon and for its dimeric S0-C_36_ structure obtained at the DFT/B3LYP (red) and the DFT/user-DFT (blue) level.

The results are shown in Figure 3. It can be seen that in both C_36_ molecules there are basically five C–C bond lengths:

- in C_36-p_: 1.49 and 1.40 in the 4-membered ring and then 1.35 and 1.23, 1.32, 1.23, etc., in the circle
- in C_36-c_: 1.51 and 1.45 in the 4-membered ring and then 1.35 and 1.25, 1.32, 1.25, etc., in the circle

Thus, in both cases the electronic structure of C_36_ is the same with the same “polyynic” bonds nature and it does not matter from what form of C_18_ (polyynic or cumulenic) we began to generate bigger structures.

### 2.2. Running Virtual Reactions with External EGO Forces

The main goal of our studies was to generate new, larger and more stable structures by connecting smaller ones starting from C_18_. The EGO method was used to induce desired reactions by pushing together selected atoms from two fragments. If the magnitude of external pushing forces was large enough, then new bonds were created. This way a new stressed structure was formed in the presence of external forces. These forces were then switched off and a stressed molecular system was relaxed, i.e., re-optimized in the standard geometry optimization procedure (SGO). This two-stage geometry optimization process, i.e., first with external forces (EGO) and then without forces (SGO), can be done in our PQS implementation in one run. Thus, starting from one energy local minimum, a molecular system is moved by external forces to another local minimum on the potential energy surface (PES) [41]. In every geometry optimization cycle, the geometry of a system and its energy is changed. Looking at the energy in consecutive optimization cycles, one can see a two-dimensional picture of the so-called algorithmic reaction path (ARP) [28,29,42,43]. Such ARPs usually exhibit at least one energy maximum. The maximum energy on the ARP corresponds to the stressed transition state (TS) which after relaxation (re-optimization without forces) gives the true TS. Differences in magnitude external forces drive a given molecular system along different ARPs and may lead to the same or different final energy local minima on PES [29,44,45].

Some of the structures presented in this paper are very complicated and it would be very difficult or even impossible to analyze the valency of the carbon atoms there. We believe that in such large molecules this issue should not be raised. The major thing is to obtain the electronic structure for a given geometrical architecture. Sometimes during geometry optimization, one may get a very strange or wrong structure for which the Hartree–Fock (HF) or Kohn–Sham (KS) equations cannot be solved, i.e., the self-consistent-field (SCF) procedure does not converge. This means that the electronic structure cannot be adjusted to that particular geometrical structure. Such molecular geometry is not approved. Otherwise, if for a given structure the SCF converges, then everything is fine regardless of the formal valency of the atoms.

### 2.3. Forming Squeezed ^S1^C_18_ and ^S2^C_18_ Molecules

These two molecules were obtained from S0-C_18_ (cyclo[18]carbon) by pushing together two atoms or two pairs of atoms from opposite sides of the C_18_ circle. The EGO algorithmic reaction paths for these two reactions are shown in Figure 1a and Figure 2a, respectively.

The first S0-C_18_$\overset{\mathrm{forces}}{\to }$S1-C_18_ reaction (Figure 1a,b) is an elementary reaction with one transition state. This TS was found after relaxation (re-optimization without forces) of the stressed TS corresponding to the energy maximum on ARP obtained with the lowest EGO forces. The energy diagram is shown in Figure 1b. It can be seen that with external forces larger in magnitude, the corresponding ARPs are always higher in energy, all the way from S0-C_18_ to S1-C_18_ and they are very similar.

The situation is more complex in the case of the second S0-C_18_$\overset{\mathrm{forces}}{\to }$S2-C_18_ reaction. It is a two-stage reaction where one C–C bond is created first, followed by the formation of the second C–C bond. This process can be seen on the animated ARP picture showing the reaction progress. The ARP plots for every external force (magnitudes: 0.075, 0.065 and 0.055 au, 1 au = ~82,400 pN) exhibit clearly two energy maxima and one energy minimum between them. Thus, there are two transition states and one local minimum. We located these stationary points. These TSs as well as the intermediate S1-C_18_ (different from S1-C_18_) are shown on the energy diagram in Figure 2b. It should also be noted that the highest energy maximum on every plot decreases with a lower magnitude of the EGO forces:

(1) $$E_{max-0.055}<E_{max-0.065}<E_{max-0.075}.$$

One important conclusion resulting from these calculations is that the maximum energy on ARP delivers an upper limit to the highest true transition state. Thus, we have a very convenient tool to study more complex multi-stage reactions even with several transition states. In such cases, finding all transition states or even the highest energy one would be very difficult, if possible at all. With our approach it is possible to estimate the upper limit for the energy demand in such complex processes. It can be seen in Figure 1b and Figure 2b that the EGO maximum energy levels (marked in red) lie above the true transition state level (marked in blue). Moreover, the maximum energy obtained with the lowest external forces gives quite an accurate estimate for the energy barriers: 83.5 versus 82.6 kcal/mol in the first reaction and 152.0 versus 145.1 for the second reaction.

**Figure 4:**
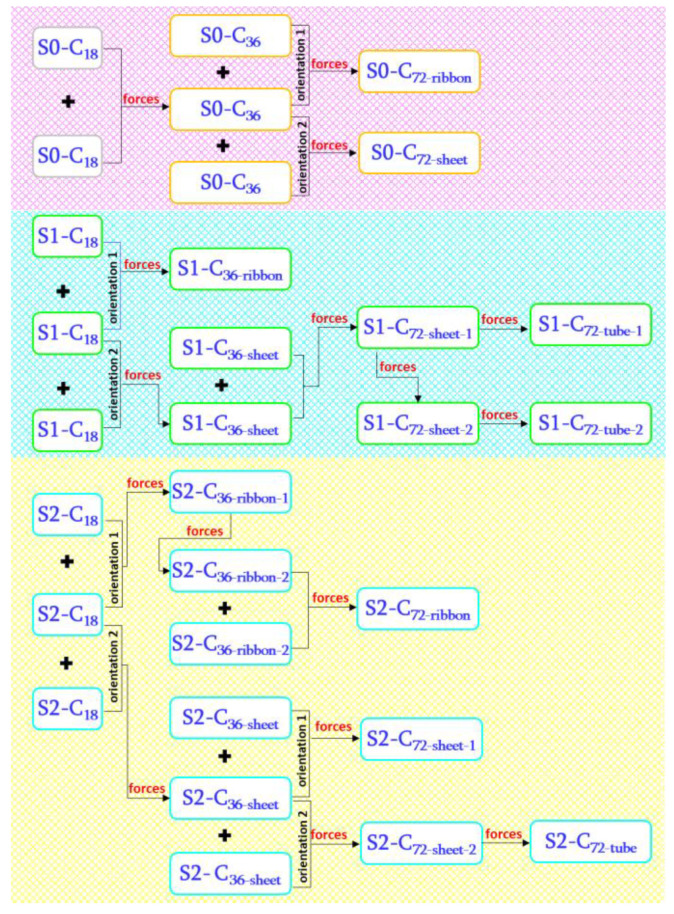
Scheme of the generation of the isomers of C_18_, C_36_ and C_72._

The energy diagrams, Figure 1b and Figure 2b, show that while the S1-C_18_ molecule should be relatively stable, the S2-C_18_ molecule with two C–C bonds will not survive and should transform back into starting S0-C_18_ in the essentially barrier-less process. Nevertheless, we used these two molecules together with S0-C_18_ to build up bigger units as shown schematically in Figure 4.

We always tried to form at least two C–C bonds between subunits to have more rigid systems. The results for every virtual reaction are presented in the energy diagrammatic form. Every diagram contains structures of the reactants, the product (marked in blue) and intermediate complexes corresponding to the energy maximum (marked in red) on ARP obtained with external forces of different magnitudes. Usually, we include three such maximum energy levels and the one obtained with the lowest external forces is used to estimate the energy barrier. However, we did not try to find the minimum external force which makes for the desired transition. Instead, we performed calculations with a given force and then two or more calculations with forces lowered by a given step size until an external force is too small to induce a reaction.

### 2.4. Forming S0-C_36_ and S0-C_72_ Molecules

**Figure 5:**
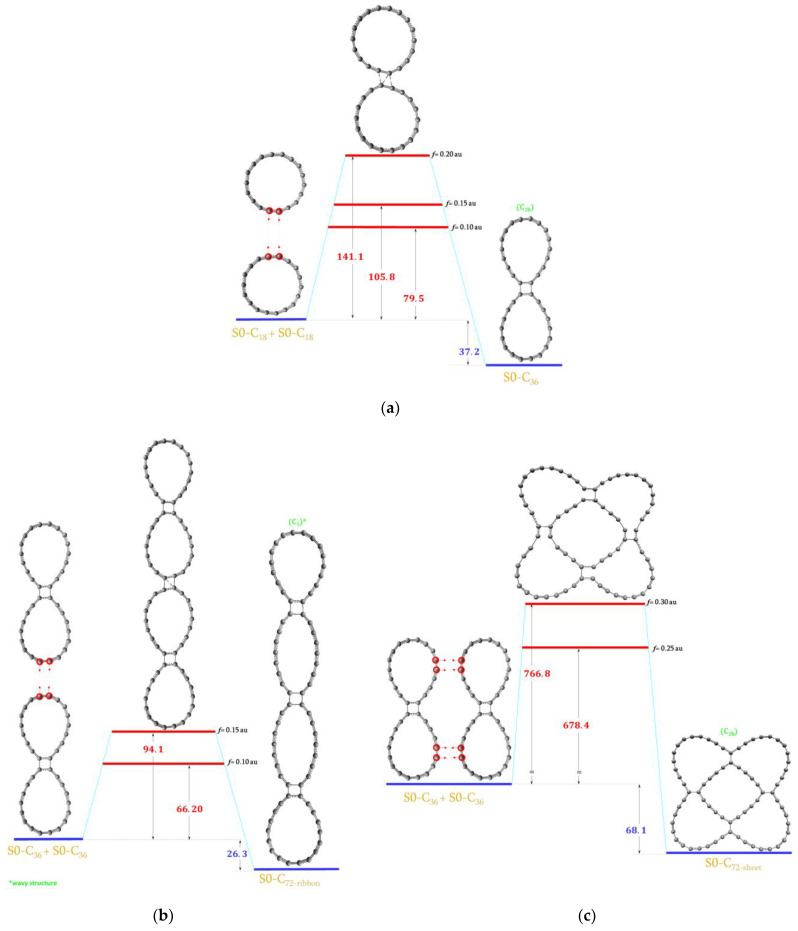
The energy diagram with the estimated upper limit of the energy demand for the formation of the (**a**) S0-C_36_ molecule; (**b**) S0-C_72-ribbon_ molecule; (**c**) S0-C_72-sheet_ molecule. All values are in kcal/mol.

Two S0-C_18_ were linked together with two C–C bonds to form the S0-C_36_ molecule. We used the EGO forces of three different magnitudes: 0.20, 0.15 and 0.10 au. The energy diagram is presented in Figure 5a. The estimated energy barrier between the reactants and the product should be less than 79.5 kcal/mol. Once S0-C_36_ is created, then it should be very stable with the estimated barrier of 116.7 kcal/mol in the reverse reaction.

In the next step, the S0-C_72-ribbon_ and S0-C_72-sheet_ molecules were generated by pushing together two S0-C_36_. This is shown in Figure 4b,c, respectively. The estimated energy demand was less than 66.2 kcal/mol to form the ribbon structure while for the sheet one, up to about 220 kcal/mol was needed. There were also significant bond rearrangements when forming the S0-C_72-sheet_ which resulted in the appearance of five 20-membered rings (Figure 5c) (two types).

### 2.5. Forming S1-C_36_ and S1-C_72_ Molecules

**Figure 6:**
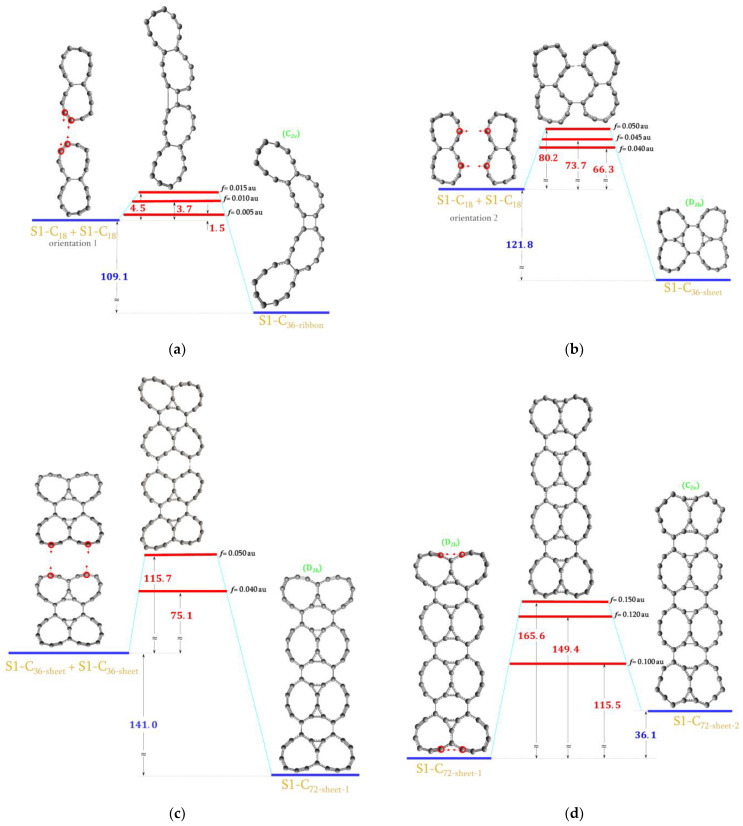

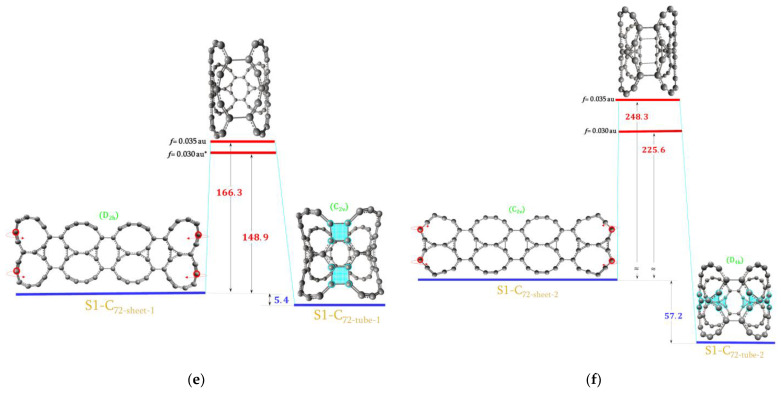
The energy diagram with the estimated upper limit of the energy demand for the formation of the (**a**) S1-C_36-ribbon_ molecule; (**b**) S1-C_36-sheet_ molecule; (**c**) S1-C_72-sheet-1_ molecule; (**d**) S1-C_72-sheet-2_ molecule; (**e**) S1-C_72-tube-1_ molecule; (**f**) S1-C_72-tube-2_ molecule. All values are in kcal/mol. * *p* < 0.05

In a similar manner starting from S1-C_18_, the series of S1-C_36_ and S1-C_72_ molecules were generated. In this case, we pushed together two ^S1^C_18_ in two different ways forming S1-C_36-ribbon_ and S1-C_36-sheet_ as shown in Figure 6a,b.

According to our estimation, it should be easy to get S1-C_36-ribbon_ (1.5 kcal/mol) while S1-C_36-sheet_ would require about 39.8 kcal/mol. Two S1-C_36-sheet_ were then linked to form S1-C_72-sheet-1_ which is presented in Figure 6c. The energy demand in this process should be less than 75.1 kcal/mol. It may be noted that each S1-C_36-sheet_ molecule consists of four 10-membered rings, one 8-membered ring and two 3-membered rings. These species are retained in the final S1-C_72-sheet-1_ as well as one more 8-membered ring and two 3-membered rings are generated in the place of bonding. However, there are no 3-membered rings at both ends which would have been beneficial if one wanted to roll this sheet up to form a tube. Thus, one more virtual reaction was performed in order to generate these “missing” 3-membered rings. This is shown in Figure 6d where the new S1-C_72-sheet-2_ molecule is also presented.

The S1-C_72__-sheet-1_ and S1-C_72-sheet-2_ molecules are wide and long enough to expect that they can be rolled up into tubes. To do so, we pushed together two pairs of atoms from opposite sides of S1-C_72_ sheets as shown in Figure 6e,f. Since both S1-C_72_ sheets are almost planar (XY), both sides of each molecule were first moved out of plane using external EGO forces directed from atoms of interest to the corresponding dummy atoms placed above the XY plane (in the XZ plane). After several optimization cycles, both sides of these molecules were out of the XY plane and external forces involving dummy atoms were switched off whilst pushing forces were applied as shown in Figure 6e,f. In both cases, two pairs of atoms were pushed together and the bonds between them were created as expected. However, when S1-C_72__- sheet-1_ was rolled, two more bonds were generated between atoms on which external forces were not applied and as a result the two 4-membered rings appeared (highlighted in Figure 6e). Both tube molecules consisted of 10-membered, 8-membered and 3-membered rings.

### 2.6. Forming S2-C_36_ and S2-C_72_ Molecules

**Figure 7:**
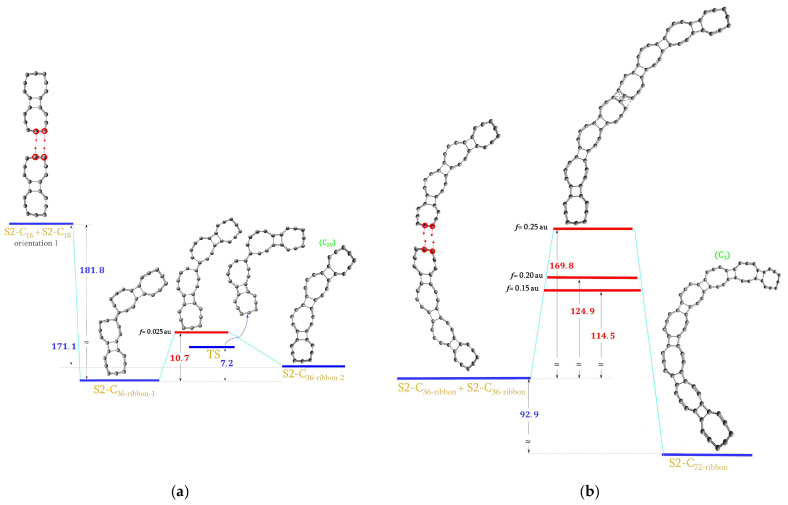

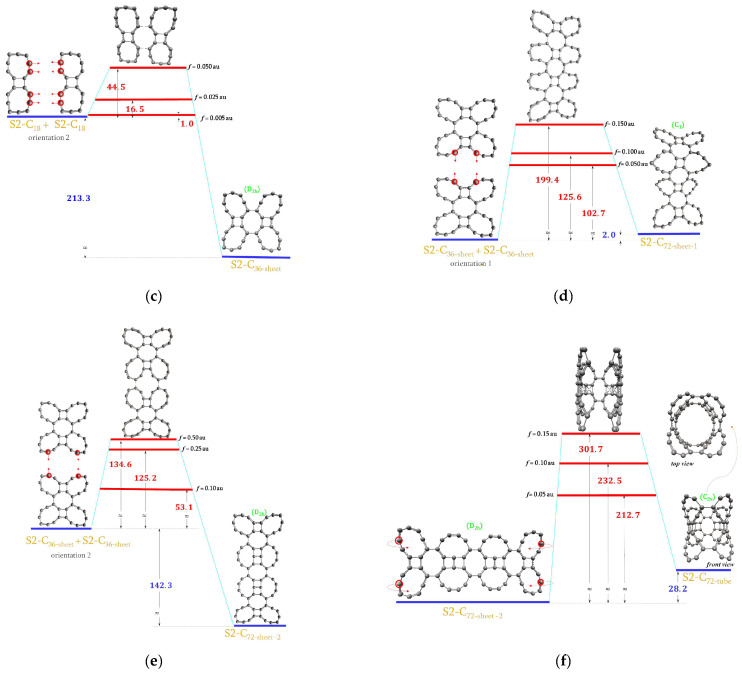
The energy diagram with the estimated upper limit of the energy demand for the formation of the (**a**) S2-C_36-ribbon-2_ molecule; (**b**) S2-C_72-ribbon_ molecule; (**c**) S2-C_36-sheet_ molecule; (**d**) S2-C_72- sheet-1_ molecule; (**e**) S2-C_72-sheet-2_ molecule; (**f**) S2-C_72-tube_ molecule. All values are in kcal/mol.

In an analogous manner, the S2-C_36_ and S2-C_72_ molecules were generated: S2-C_36-ribbon-2_ and S2-C_72-ribbon_ as shown in Figure 7a,b, as well as S2-C_36-sheet_, S2-C_72-sheet- 1_ and S2-C_72-sheet-2_ shown in Figure 7c,d,e. Virtual reactions leading to these structures were quite complex. However, one reaction goes differently from others. So far, in all the virtual reactions presented here, the energy of a system was always first increasing when external pushing forces were applied to two reactants and then it was decreasing after the energy maximum was reached. In the case of the S2-C_18_ + S2-C_18_→S2-C_36-ribbon-2_ reaction, the energy first goes drastically down and the intermediate ^S2^C_36-ribbon-1_ is created spontaneously. This structure corresponds to the energy local minimum. In this case, we were able to locate the transition state linking both S2-C_36_ ribbons. This is shown in Figure 7a. It can be seen that our estimated energy barrier of 10.7 kcal/mol (red) is quite close to the real energy barrier of 7.2 kcal/mol (blue). Much higher energy, up to 114.5 kcal/mol, is needed to generate the S2-C_72-ribbon_ molecule as shown in Figure 7b.

As discussed before, it was quite easy to link together two S2-C_18_ to form S2-C_36- ribbon_ and again it was easy to get S2-C_36-sheet_ as shown in Figure 7c. In this case, we wanted to create four C–C bonds in order to get two more 4-membered rings. However, only two new bonds were formed in the S2-C_36-sheet_. Two of these molecules were then linked in two ways by applying external forces to different atoms as shown in Figure 7d,e. As a result, two different S2-C_72_ sheets were obtained: S2-C_72-sheet-1_ (wavy) and S2-C_72-sheet-2_ with new moieties, i.e., two connected 4-membered rings. The S2-C_72-sheet-2_ molecule was then rolled up to form a tube, as is presented in Figure 7f. The energy demand to form S2-C_72-tube_ was very high just as before in the case of S1-C_72-tube-1_ and S1-C_72-tube-2_.

In the Supporting Information Section, the absolute energies, lowest vibrational frequencies and symmetry info are enclosed in Table S1 for all stationary points reported here. In Table S2, atomic coordinates are given. There is also a link to the website page where the animated movies of all the virtual reactions can be seen.

### 2.7. Energy Requirement

Running virtual reactions driven by external forces, we generated nineteen structures corresponding to the energy local minimum and four structures corresponding to the saddle points on PESs. The energy demand for every reaction was also estimated. This allowed us to assess both the possibility of forming a given structure and also its stability. In most cases, the energy demand was high or even very high; thus, corresponding structures could not be obtained spontaneously by increasing the temperature. On the other hand, the energy barriers for reverse reactions were also high which means that these structures once obtained will be stable. Some of them have low vibrational frequencies (see Table S1) which means a shallow energy minimum on PES. This may, but not necessarily, indicate instability.

The high energy demand to form most of the compounds presented here does not exclude that they can be obtained in other reactions using catalysts or the AFM type techniques which is most promising. This way the cyclo[18]carbon was synthesized from the cyclocarbon oxide molecule [39] by removing two, four or six CO molecules. We examined these reactions using the EGO pulling forces to break C–C bonds. We considered two ways: removing all CO at once,

C_24_O_6_—6 CO → C_18_

and step by step.

C_24_O_6_—2 CO → C_22_O_4_

C_22_O4—2 CO → C_20_O_2_

C_20_O_2_—2 CO → C_18_

The obtained results for the energy demands were very interesting: the estimated energy barrier for the first reaction was about 220 kcal/mol, while removing two CO at once requires about 71, 113 and 94 kcal/mol in three steps, respectively. It shows that the energy barriers to form C_18_ from C_24_O_6_ are also high; however, in spite of them, it was still possible to get cyclo[18]carbon using the AFM techniques.

### 2.8. HOMO–LUMO Gap

The energies of the HOMO and LUMO frontier orbitals were often used in chemistry to describe and interpret a wide range of chemical interactions. These energies, along with the values of related quantities such as ionization potential IA, electron affinity EA, chemical potential ě, global hardness ç and global electrophilicity index ω, can give an insight into the properties and reactivity of a molecule [46]. For the starting cyclo[18]carbon molecule and for eighteen pure-carbon nanostructures of various shapes presented in this work, these data are summarized in Table 1. As can be seen, these nanostructures are characterized by amazingly low HOMO–LUMO energy gap values even below 0.5 eV! Such extremely low HOMO–LUMO gaps can be associated with many unusual electronic properties and may open completely new possible applications [47]. This makes them ideal models for the fundamental study of electron-transfer phenomena as well as potential objects for molecular electronics [48,49].

It can be seen that a *simple* modification of the starting system (C_18_) related to either compression (S1-C_18_) or dimerization (S0-C_36_) leads to a substantial reduction of the HOMO–LUMO gap (3.13 vs. 1.57 and 2.97 eV, respectively). Overall, the HOMO–LUMO gap turns out to be strictly *size-dependent*. Doubling the size of the molecular system roughly halves the HOMO–LUMO gap energy (e.g., for the S1 series: ~3 eV for S1C_18_, ~1.7 to S1C_36sheet_ and finally ~0.70 eV for S1C_72sheet-1_). This is due to the increase in the HOMO and the simultaneous decrease in the LUMO energy. Moreover, for a given molecular size, the HOMO–LUMO energy gap also depends on molecular shape. For the S0 and S1 series, the HOMO–LUMO gaps for ribbon-like systems are substantially higher than for sheet-like molecular systems. Interestingly, a reverse trend can be observed for the S2 series.

The unique semiconductor property of the cyclo[18]carbon and its derivatives makes them possible elements for molecular electronic devices. The very first theoretical works on the properties of systems based on the cyclic C_18_ structure can be found in the literature [50,51].

Additionally, the HOMO and LUMO energies and related quantities (IA, EA, ç, ě, ů) are important descriptors for chemical reactivity. The large HOMO–LUMO gap may be responsible for low chemical reactivity and high kinetic stability, while the small HOMO–LUMO gap indicates low chemical stability. This is because removing electrons from the low-lying HOMO or adding electrons to the high-lying LUMO is more energetically preferred in any chemical reaction. Likewise, an increase in the global chemical hardness can lead to a more stable configuration. It is related to the resistance to deformation or polarization of the electron cloud of molecules. According to the HOMO–LUMO gap and the chemical hardness values, the starting cyclic C_18_ structure and S1-C_18_, S1-C_36-ribbon_ and S2-C_36- sheet_ are the most stable systems. In turn, large softness values indicate high chemical reactivity. Softness values for the majority of the presented molecules oscillate around 1 eV, which suggests their high reactivity.

There is one important issue regarding the HOMO–LUMO gap and the SCF convergence in the calculations presented here. The HOMO–LUMO gap in all C_18_, C_36_ and C_72_ molecules which is very small (for instance: 0.1152 au in S0-C_18_, 0.0059 au in S0-C_72-sheet_) slows down the SCF convergence. Sometimes during geometry optimization, the geometrical change enforces a large change in the electronic structure which involves significant bond re-arrangement. In such a case, the SCF convergence is very slow (requiring even ~50 iterations). Moreover, depending on the SCF initial guess, it may converge to different electronic states. In our calculations, all molecules were treated as the singlet states of the closed-shell systems.

## 3. Materials and Methods

All calculations were performed at the density functional theory (DFT) [52,53] level with the B3LYP [54] exchange-correlation potential, using the 6-31g-dp basis set [55,56]. Calculations were done in parallel mode with the PQS program package [57] and obtained molecular structures were analyzed (visualized) using our Graphical User Interface, PQSMol. The nature of every final structure found in these studies was verified by the vibrational analysis and only structures corresponding to energy local minima on potential energy surfaces (PES) are reported here.

## 4. Conclusions

In the present studies, the EGO method was used to run virtual reactions where two smaller subsystems were linked together to form a bigger system. This way, starting from C_18_, we generated a series of C_36_ and C_72_ novel structures of different types including ribbons, sheets and tubes. There are three new isomers of C_18_, six isomers of C_32_ and ten isomers of C_72_.

Two isomers of C_18_, namely S1-C_18_ and S2-C_18_, have been found before [14]. The major difference between those and our findings is how these structures have been obtained. Those have, in fact, simply been invented, i.e., deduced or guessed based upon the carbon valency (just like Kekule’s structure of benzene), while ours have been obtained via virtual reactions. There is no way one could guess or deduce the existence of some bigger structures such as, for instance, S2-C_72-sheet_ or others. Moreover, our procedure could be continued and many more C_36_ and C_72_ isomers could be generated by linking different C_18_ and C_36_ units, for instance mixing S0, S1 and S2 structures.

The most important outcomes of our studies are the novel C**_n_** structures generated from the cyclo[18]carbon molecule, mostly in regards to their shape. We think that some of the molecular structures shown here are highly impressive. In some cases, the bonding networks that exist are just amazing. There are a variety of moieties: 3-, 4-, 8-, 9-, 10-, 18- and 20-membered rings, as well as even 11-membered ring in C^’^_18_ (intermediate in Figure 2d). It is also very worthwhile to see how the new bonds are formed and how others are rearranged during enforced reactions. Every reaction presented in this paper can be viewed as an animated movie (please check the relevant materials in the Supplementary Information Section). We encourage readers to see the motion pictures of these reactions.

The carbon nanostructures have been studied as very promising systems in many areas of nanotechnology and nanomedicine. These nanostructures can be divided into several groups such as: graphene and graphene oxide, carbon nanotubes, carbon nanohorns and nano-onions, nanodiamond or carbon quantum dots. Generally, they differ from each other in spatial structure, dimension and organization of bonds between carbon atoms. These structures are usually considered as well-defined ones, but it is obvious that they are always terminated in some way. That termination area might differ significantly from the bulk/spatial structure and sometimes it may even govern the overall chemistry of the whole nanostructure. Therefore, understanding of such little defined or disordered carbon structures is crucial for the analysis of the physicochemical properties of actual structures such as, e.g., carbon quantum dots with copious unusual surface carbon linkages on their surfaces and functional groups. We believe that the structures obtained from cyclo[18]carbon may be present in the terminal areas of various carbonaceous materials. According to our previous experience with the analysis of fracture phenomena of carbon nanotubes using reactive empirical bond-order potential (ai-REBO) [58,59], we can expect that the newly created surfaces of carbonaceous materials are very rich in non-stoichiometric lineages between carbon atoms [60]. That conclusion is not surprising since SEM/TEM images of amorphous carbons or even formally well-defined carbon nanostructures are locally, in the atomic length scale, highly corrugated. The current study provides evidence that the existence of such exceptional linkages can be proved on rigorous quantum chemical theoretical levels. Therefore, the described chemistry of cyclo[18]carbon may also be representative of carbonaceous nanomaterials extensively studied as therapeutic and diagnostic agents in medicine and pharmacology.

## Figures and Tables

**Table 1:** The HOMO and LUMO energies and related quantities (all values in eV).

Structure	E_H_	E_L_	ΔE_H-L_	IP	EA	μ	η	s	ω
C_18_	−6.313	−3.184	3.129	6.313	3.184	4.748	1.565	0.639	0.258
S0-C_36_	−5.552	−3.981	1.572	5.552	3.981	4.767	0.786	1.272	0.065
S0-C_72-sheet_	−5.348	−4.575	0.773	5.348	4.575	4.962	0.387	2.586	0.015
S0-C_72-ribbon_	−5.338	−4.344	0.994	5.338	4.344	4.841	0.497	2.013	0.025
S1-C_18_	−6.093	−3.119	2.974	6.093	3.119	4.606	1.487	0.672	0.240
S1-C_36-ribbon_	−5.580	−3.650	1.931	5.580	3.650	4.615	0.965	1.036	0.101
S1-C_36-sheet_	−5.450	−3.754	1.696	5.450	3.754	4.602	0.848	1.179	0.078
S1-C_72-sheet−1_	−4.960	−4.263	0.696	4.960	4.263	4.612	0.348	2.873	0.013
S1-C_72-sheet−2_	−5.476	−4.550	0.926	5.476	4.550	5.013	0.463	2.160	0.021
S1-C_72-tube−1_	−4.843	−3.730	1.113	4.843	3.730	4.286	0.556	1.797	0.036
S1-C_72-tube−2_	−5.024	−4.191	0.833	5.024	4.191	4.607	0.416	2.402	0.019
S2-C_18_	−6.198	−5.809	0.389	6.198	5.809	6.003	0.194	5.143	0.003
S2-C_36-ribbon−1_	−6.381	−4.739	1.642	6.381	4.739	5.560	0.821	1.218	0.061
S2-C_36-ribbon−2_	−6.180	−5.458	0.722	6.180	5.458	5.819	0.361	2.768	0.011
S2-C_36-sheet_	−5.663	−3.802	1.861	5.663	3.802	4.733	0.930	1.075	0.091
S2-C_72-ribbon_	−5.857	−5.420	0.437	5.857	5.420	5.638	0.219	4.574	0.004
S2-C_72-sheet1_	−5.724	−4.605	1.119	5.724	4.605	5.164	0.560	1.787	0.030
S2-C_72-sheet2_	−5.272	−3.865	1.407	5.272	3.865	4.569	0.704	1.421	0.054
S2-C_72-tube_	−5.318	−3.806	1.512	5.318	3.806	4.562	0.756	1.323	0.063

Μ—chemical potential, η—global chemical hardness, s—global softness, ω—global electrophilicity index, IP—ionization potential, EA—electron affinity.

## Data Availability

The data that support the findings of this study are available from the authors.

## Supplementary Materials

The following supporting information can be downloaded at: https://www.mdpi.com/article/10.3390/ijms232112960/s1.
