# Supplementary material for: From Cyclo[18]carbon to the Novel Nanostructures—Theoretical Predictions
Source: Int J Mol Sci. 2022 Oct 26;23(21):12960. doi: 10.3390/ijms232112960 (PMC9654130; doi:10.3390/ijms232112960)
Supplement: Supplementary file 1 [file ijms-23-12960-s001.zip › ijms-1971282-supplementary.pdf]

International Journal of
Molecular Sciences

MDPI

Supplementary Information

From Cyclo[18]carbon to the Novel Nanostructures. Theoretical Predictions.

Agnieszka Brzyska[*], *Tomasz Panczyk*[1*]
and Krzysztof Wolinski[†]

[*]Jerzy Haber Institute of Catalysis and Surface Chemistry, Polish Academy of Sciences, Niezapominajek 8, 30-239 Krakow, Poland ; [1]Corresponding author: tpanczyk95@gmail.com

†Department of Theoretical Chemistry, Institute of Chemical Sciences, Faculty of Chemistry, Maria Curie Sklodowska University in Lublin, pl. Maria Curie-Sklodowska 3, 20-031 Lublin, Poland

Table S1. The energy and the lowest vibrational frequency values obtained at the DFT/B3LYP/6-31g-dp level for the isomers of C_{18}, C_{36} and C_{72}.

Structure	E[au]	ν_{min}[cm^{-1}]	Symmetry
$^{S0}C_{18}$	-685.358037	60.8	D_{18h}
$^{S0}C_{18}$(TS)	-685.226415	-454.5	C_{2v}
$^{S0}C_{36}$	-1370.7748909	19.7	C_{2h}
$^{S0}C_{72\text{-ribbon}}$	-2741.592654	12.8	C_1
$^{S0}C_{72\text{-sheet}}$	-2741.656951	14.2	C_{2h}
$^{S1}C_{18}$	-685.2475122	54.16	C_{2v}
$^{S1}C_{36\text{-ribbon}}$	-1370.6688861	22.4	C_{2v}
$^{S1}C_{36\text{-sheet}}$	-1370.6890768	25.4	D_{2h}
$^{S1}C_{72\text{-sheet-1}}$	-2741.602896	14.9	D_{2h}
$^{S1}C_{72\text{-sheet-2}}$	-2741.545390	15.3	C_{2v}
$^{S1}C_{72\text{-tube-1}}$	-2741.611567	39.6	C_{2v}
$^{S1}C_{72\text{-tube-2}}$	-2741.636157	36.4	C_{2h}
$^{S2}C_{18}$	-685.128517	37.7	C_{2v}

$^{S2}C_{18}$(TS1)	-685.198184	-190.3	C_2
$^{S2}C_{18\text{-int}}$	-685.200911	46.4	C_2
$^{S2}C_{18}$(TS2)	-685.126754	-519.1	C_{2v}
$^{S2}C_{36\text{-ribbon-1}}$	-1370.546734	17.25	C_{2v}
$^{S2}C_{36\text{-ribbon-1}}$(TS)	-1370.518246	-317.9	C_{2v}
$^{S2}C_{36\text{-ribbon-2}}$	-1370.529725	13.0	C_{2v}
$^{S2}C_{72\text{-ribbon}}$	-2741.207540	13.6	D_{2h}
$^{S2}C_{36\text{-sheet}}$	-1370.596843	47.4	C_1
$^{S2}C_{72\text{-sheet-1}}$	-2741.190539	25.7	D_{2h}
$^{S2}C_{72\text{-sheet-2}}$	-2741.420583	10.6	C_1
$^{S2}C_{72\text{-tube}}$	-2741.375611	38.4	C_{2v}

Table S2. The Cartesian coordinates (X,Y,Z) for the generated structures (C18, C36 and C72).

**1.**	$^{S0}C_{18}$				
	1 c	0.224210	-3.728802	0.000000	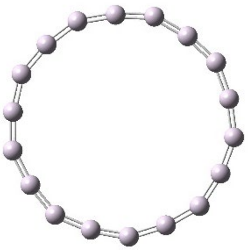
	2 c	-1.040533	-3.497843	0.000000	
	3 c	1.451361	-3.348215	0.000000	
	4 c	-2.225058	-3.000328	0.000000	
	5 c	2.568959	-2.712636	0.000000	
	6 c	-3.045804	-2.010777	0.000000	
	7 c	3.263843	-1.631967	0.000000	
	8 c	-3.633496	-0.868241	0.000000	
	9 c	3.711738	-0.426827	0.000000	
	10 c	-3.625910	0.417344	0.000000	
	11 c	3.549136	0.847715	0.000000	
	12 c	-3.341465	1.670300	0.000000	
	13 c	3.117713	2.058810	0.000000	
	14 c	-2.509121	2.650066	0.000000	
	15 c	2.174016	2.930783	0.000000	
	16 c	-1.485931	3.427210	0.000000	
	17 c	1.064866	3.580815	0.000000	
	18 c	-0.218524	3.642595	0.000000	
**2.**	$^{S0}C_{36}$				
	1 c	-5.091829	-3.326623	0.000000	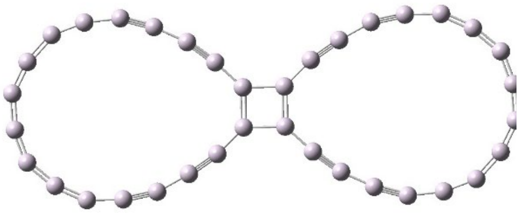
	2 c	-6.405348	-3.354966	0.000000	
	3 c	-3.865552	-3.053551	0.000000	
	4 c	-7.554743	-2.839837	0.000000	
	5 c	-2.716232	-2.411617	0.000000	
	6 c	-8.569416	-2.006459	0.000000	
	7 c	-1.690713	-1.695883	0.000000	
	8 c	-9.011327	-0.824660	0.000000	

	9	c	-0.737340	-0.739401	0.000000	
	10	c	-9.107449	0.483486	0.000000	
	11	c	-0.766816	0.704686	0.000000	
	12	c	-8.557438	1.619659	0.000000	
	13	c	-1.767978	1.608555	0.000000	
	14	c	-7.679879	2.592895	0.000000	
	15	c	-2.785022	2.337439	0.000000	
	16	c	-6.501596	3.041925	0.000000	
	17	c	-3.982249	2.877798	0.000000	
	18	c	-5.200108	3.193023	0.000000	
	19	c	5.200108	-3.193023	0.000000	
	20	c	3.982249	-2.877798	0.000000	
	21	c	6.501596	-3.041925	0.000000	
	22	c	2.785022	-2.337439	0.000000	
	23	c	7.679879	-2.592895	0.000000	
	24	c	1.767978	-1.608555	0.000000	
	25	c	8.557438	-1.619659	0.000000	
	26	c	0.766816	-0.704686	0.000000	
	27	c	9.107449	-0.483486	0.000000	
	28	c	0.737340	0.739401	0.000000	
	29	c	9.011327	0.824660	0.000000	
	30	c	1.690713	1.695883	0.000000	
	31	c	8.569416	2.006459	0.000000	
	32	c	2.716232	2.411617	0.000000	
	33	c	7.554743	2.839837	0.000000	
	34	c	3.865552	3.053551	0.000000	
	35	c	6.405348	3.354966	0.000000	
	36	c	5.091829	3.326623	0.000000	
3.	$^{S0}C_{72\text{-ribbon}}$					

	1	c	-15.919655	-3.855167	0.304360	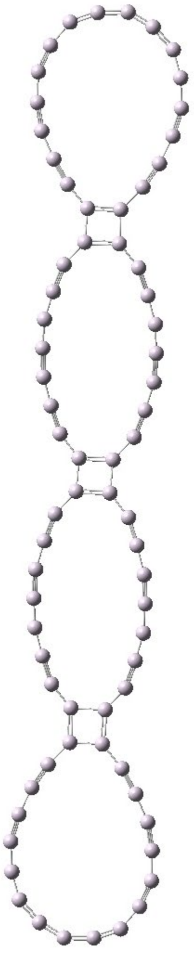
	2	c	-17.235360	-3.920404	0.219376	
	3	c	-14.703013	-3.570354	0.368167	
	4	c	-18.404526	-3.478343	0.113315	
	5	c	-13.535052	-2.949641	0.384146	
	6	c	-19.469424	-2.708414	-0.007941	
	7	c	-12.493314	-2.266152	0.362120	
	8	c	-19.971963	-1.561636	-0.117811	
	9	c	-11.537506	-1.304951	0.245334	
	10	c	-20.104782	-0.253039	-0.209895	
	11	c	-11.607322	0.133057	0.164650	
	12	c	-19.558346	0.879318	-0.242591	
	13	c	-12.656030	0.997746	0.157569	
	14	c	-18.655819	1.838132	-0.243861	
	15	c	-13.724526	1.636054	0.095952	
	16	c	-17.472404	2.256271	-0.190939	
	17	c	-14.949454	2.118525	-0.005778	
	18	c	-16.166061	2.397996	-0.108813	
	19	c	-5.264403	-2.710935	0.512658	
	20	c	-6.547426	-2.721727	0.394982	
	21	c	-4.000560	-2.580138	0.603802	
	22	c	-7.795264	-2.445024	0.266590	
	23	c	-2.766844	-2.171438	0.622831	
	24	c	-9.018703	-2.093040	0.159072	
	25	c	-1.596723	-1.694670	0.582483	
	26	c	-10.061557	-1.278790	0.081462	
	27	c	-0.633092	-0.773564	0.414443	
	28	c	-10.135328	0.236035	0.000021	
	29	c	-0.711637	0.707725	0.386566	
	30	c	-9.182291	1.156967	-0.005600	
	31	c	-1.769677	1.529646	0.496134	
	32	c	-8.001047	1.639127	0.072710	
	33	c	-2.979130	1.895717	0.482499	
	34	c	-6.790354	2.054866	0.177531	
	35	c	-4.245221	2.184366	0.415141	
	36	c	-5.513871	2.181100	0.302106	
	37	c	5.546579	-2.236290	0.050679	
	38	c	4.275169	-2.221977	-0.087250	
	39	c	6.818843	-2.127520	0.196996	
	40	c	3.018416	-1.917748	-0.167342	
	41	c	8.035424	-1.715660	0.309816	
	42	c	1.808688	-1.536963	-0.185761	
	43	c	9.209228	-1.224210	0.376262	
	44	c	0.759403	-0.713910	-0.082912	

	45	c	10.166374	-0.301891	0.371155	
	46	c	0.683218	0.777582	-0.097436	
	47	c	10.089221	1.200799	0.430860	
	48	c	1.649446	1.699353	-0.187595	
	49	c	9.042028	2.017188	0.492482	
	50	c	2.820806	2.182865	-0.133051	
	51	c	7.828970	2.400673	0.432831	
	52	c	4.044987	2.590953	-0.016654	
	53	c	6.581261	2.703689	0.315808	
	54	c	5.307463	2.708376	0.147732	
	55	c	16.150210	-2.348167	-0.760010	
	56	c	14.931971	-2.090580	-0.622251	
	57	c	17.452043	-2.188273	-0.878776	
	58	c	13.704671	-1.632558	-0.456982	
	59	c	18.628199	-1.756440	-0.967451	
	60	c	12.634129	-1.023837	-0.262685	
	61	c	19.523664	-0.790510	-0.989416	
	62	c	11.601319	-0.179364	-0.000236	
	63	c	20.064420	0.344935	-0.959678	
	64	c	11.528661	1.260492	0.066209	
	65	c	19.924506	1.652880	-0.860096	
	66	c	12.460704	2.238523	-0.094875	
	67	c	19.411390	2.794826	-0.743680	
	68	c	13.484733	2.941614	-0.189704	
	69	c	18.337399	3.549267	-0.600545	
	70	c	14.639983	3.581278	-0.271993	
	71	c	17.163449	3.970930	-0.468742	
	72	c	15.850709	3.884455	-0.358501	
**4.**	$^{S0}C_{72\text{-sheet}}$					
	1	c	-5.232917	0.612095	0.000000	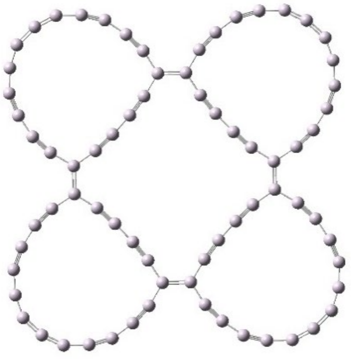
	2	c	-6.435784	1.308533	0.000000	
	3	c	-4.097201	1.400438	0.000000	
	4	c	-7.362765	2.130840	0.000000	
	5	c	-3.196202	2.254206	0.000000	
	6	c	-8.109262	3.228025	0.000000	
	7	c	-2.309285	3.244402	0.000000	
	8	c	-8.577069	4.385188	0.000000	
	9	c	-1.565460	4.238015	0.000000	
	10	c	-8.554127	5.708210	0.000000	
	11	c	-0.855757	5.423735	0.000000	
	12	c	-8.186921	6.904217	0.000000	
	13	c	-1.607299	6.594062	0.000000	
	14	c	-7.253435	7.842923	0.000000	

	15	c	-2.501606	7.450813	0.000000	
	16	c	-6.149012	8.429887	0.000000	
	17	c	-3.606073	8.193381	0.000000	
	18	c	-4.824294	8.461113	0.000000	
	19	c	5.193423	0.848751	0.000000	
	20	c	4.016606	1.573073	0.000000	
	21	c	6.357020	1.609831	0.000000	
	22	c	3.081195	2.388673	0.000000	
	23	c	7.242546	2.477044	0.000000	
	24	c	2.151914	3.338240	0.000000	
	25	c	7.941742	3.604961	0.000000	
	26	c	1.363655	4.296633	0.000000	
	27	c	8.356831	4.782291	0.000000	
	28	c	0.607004	5.453137	0.000000	
	29	c	8.267669	6.101941	0.000000	
	30	c	1.306772	6.654832	0.000000	
	31	c	7.857589	7.283649	0.000000	
	32	c	2.155189	7.556685	0.000000	
	33	c	6.884119	8.180245	0.000000	
	34	c	3.224094	8.349408	0.000000	
	35	c	5.751565	8.709899	0.000000	
	36	c	4.427404	8.676318	0.000000	
	37	c	-4.427404	-8.676318	0.000000	
	38	c	-5.751565	-8.709899	0.000000	
	39	c	-3.224094	-8.349408	0.000000	
	40	c	-6.884119	-8.180245	0.000000	
	41	c	-2.155189	-7.556685	0.000000	
	42	c	-7.857589	-7.283649	0.000000	
	43	c	-1.306772	-6.654832	0.000000	
	44	c	-8.267669	-6.101941	0.000000	
	45	c	-0.607004	-5.453137	0.000000	
	46	c	-8.356831	-4.782291	0.000000	
	47	c	-1.363655	-4.296633	0.000000	
	48	c	-7.941742	-3.604961	0.000000	
	49	c	-2.151914	-3.338240	0.000000	
	50	c	-7.242546	-2.477044	0.000000	
	51	c	-3.081195	-2.388673	0.000000	
	52	c	-6.357020	-1.609831	0.000000	
	53	c	-4.016606	-1.573073	0.000000	
	54	c	-5.193423	-0.848751	0.000000	
	55	c	4.824294	-8.461113	0.000000	
	56	c	3.606073	-8.193381	0.000000	
	57	c	6.149012	-8.429887	0.000000	
	58	c	2.501606	-7.450813	0.000000	
	59	c	7.253435	-7.842923	0.000000	
	60	c	1.607299	-6.594062	0.000000	
	61	c	8.186921	-6.904217	0.000000	
	62	c	0.855757	-5.423735	0.000000	
	63	c	8.554127	-5.708210	0.000000	
	64	c	1.565460	-4.238015	0.000000	
	65	c	8.577069	-4.385188	0.000000	

	66 c 2.309285 -3.244402 0.000000<br>67 c 8.109262 -3.228025 0.000000<br>68 c 3.196202 -2.254206 0.000000<br>69 c 7.362765 -2.130840 0.000000<br>70 c 4.097201 -1.400438 0.000000<br>71 c 6.435784 -1.308533 0.000000<br>72 c 5.232917 -0.612095 0.000000	
**5.**	$^{S0}C_{18}$ (TS)	
	1 c 0.056786 -0.945232 0.000044<br>2 c -1.114265 -1.581247 0.000093<br>3 c 1.295098 -1.436595 -0.000067<br>4 c -2.308012 -1.997826 0.000139<br>5 c 2.530368 -1.707727 -0.000158<br>6 c -3.582086 -1.712738 0.000140<br>7 c 3.760698 -1.272071 -0.000166<br>8 c -4.661339 -1.022423 0.000096<br>9 c 4.750626 -0.458043 -0.000111<br>10 c -4.526103 0.292977 -<br>0.000056<br>11 c 4.458053 0.831132 0.000069<br>12 c -3.907436 1.403868 -<br>0.000178<br>13 c 3.711576 1.860964 0.000211<br>14 c -2.590720 1.575907 -<br>0.000147<br>15 c 2.384137 1.874220 0.000160<br>16 c -1.335783 1.517916 -<br>0.000093<br>17 c 1.144665 1.667348 0.000073<br>18 c -0.066263 1.109571 -<br>0.000050	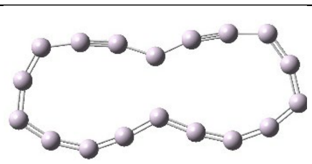
**6.**	$^{S1}C_{18}$	
	1 c 0.040397 -0.673627 0.000000<br>2 c -1.106155 -1.456587 0.000000<br>3 c 1.272396 -1.313737 0.000000<br>4 c -2.240352 -1.964862 0.000000<br>5 c 2.459340 -1.682465 0.000000<br>6 c -3.552517 -1.737710 0.000000<br>7 c 3.734829 -1.299822 0.000000<br>8 c -4.600823 -1.036310 0.000000<br>9 c 4.691938 -0.478274 0.000000<br>10 c -4.501763 0.304095 0.000000<br>11 c 4.432740 0.840556 0.000000<br>12 c -3.883510 1.396973 0.000000<br>13 c 3.688609 1.851936 0.000000<br>14 c -2.537750 1.564534 0.000000<br>15 c 2.332421 1.856763 0.000000<br>16 c -1.308217 1.427889 0.000000<br>17 c 1.128019 1.574144 0.000000<br>18 c -0.049602 0.826504 0.000000	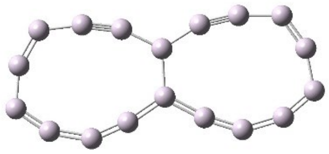

**7.**	$^{S1}C_{36\text{-ribbon}}$	
	1 c -4.272835 -0.461607 0.000000<br>2 c -4.495305 -1.836873 0.000000<br>3 c -2.974580 0.019335 0.000000<br>4 c -4.916130 -3.004344 0.000000<br>5 c -1.850848 0.542956 0.000000<br>6 c -5.988997 -3.797815 0.000000<br>7 c -0.791062 1.401203 0.000000<br>8 c -7.217133 -4.078264 0.000000<br>9 c -0.820282 2.829164 0.000000<br>10 c -8.123263 -3.082722 0.000000<br>11 c -1.966898 3.572448 0.000000<br>12 c -8.504503 -1.887172 0.000000<br>13 c -3.209939 3.444228 0.000000<br>14 c -7.698589 -0.793775 0.000000<br>15 c -4.389223 2.833014 0.000000<br>16 c -6.763303 0.015061 0.000000<br>17 c -5.107014 1.819253 0.000000<br>18 c -5.446603 0.479510 0.000000<br>19 c 4.288915 -0.261581 0.000000<br>20 c 2.969716 0.158439 0.000000<br>21 c 4.575753 -1.624913 0.000000<br>22 c 1.822711 0.628909 0.000000<br>23 c 5.053490 -2.770183 0.000000<br>24 c 0.723949 1.436681 0.000000<br>25 c 6.162403 -3.512607 0.000000<br>26 c 0.685873 2.864492 0.000000<br>27 c 7.402601 -3.732528 0.000000<br>28 c 1.796201 3.660956 0.000000<br>29 c 8.262005 -2.696405 0.000000<br>30 c 3.043883 3.590701 0.000000<br>31 c 8.584419 -1.483808 0.000000<br>32 c 4.250455 3.035324 0.000000<br>33 c 7.728005 -0.429603 0.000000<br>34 c 5.014763 2.056143 0.000000<br>35 c 6.754287 0.332471 0.000000<br>36 c 5.417079 0.733911 0.000000	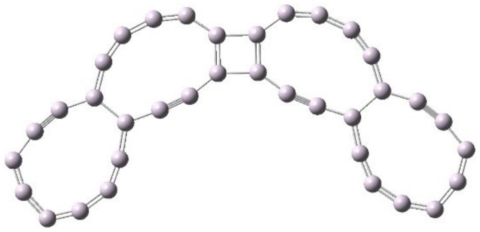
**8.**	$^{S1}C_{36\text{-sheet}}$	

	1 c 0.127400 -4.136231 0.000000<br>2 c -1.085366 -4.830908 0.000000<br>3 c 1.381737 -4.752502 0.000000<br>4 c -2.286989 -5.114687 0.000000<br>5 c 2.597810 -4.966035 0.000000<br>6 c -3.610097 -4.834168 0.000000<br>7 c 3.900878 -4.603181 0.000000<br>8 c -4.154424 -3.709434 0.000000<br>9 c 4.375831 -3.447522 0.000000<br>10 c -3.943297 -2.388062<br>0.000000<br>11 c 4.081287 -2.142125 0.000000<br>12 c -3.047525 -1.530138<br>0.000000<br>13 c 3.134429 -1.341015 0.000000<br>14 c -1.863345 -0.797452<br>0.000000<br>15 c 1.907710 -0.681974 0.000000<br>16 c -0.670956 -1.512321<br>0.000000<br>17 c 0.761032 -1.467963 0.000000<br>18 c 0.082508 -2.700212 0.000000<br>19 c -0.083208 2.699557 0.000000<br>20 c -0.761840 1.467365 0.000000<br>21 c 0.670117 1.511569 0.000000<br>22 c -1.908625 0.681485 0.000000<br>23 c 1.862606 0.797014 0.000000<br>24 c -3.135457 1.340592 0.000000<br>25 c 3.046792 1.529845 0.000000<br>26 c -4.080041 2.144096 0.000000<br>27 c 3.941910 2.388370 0.000000<br>28 c -4.378557 3.448915 0.000000<br>29 c 4.155139 3.709449 0.000000<br>30 c -3.897808 4.601918 0.000000<br>31 c 3.610271 4.833828 0.000000<br>32 c -2.596350 4.968735 0.000000<br>33 c 2.287376 5.115852 0.000000<br>34 c -1.380602 4.752933 0.000000<br>35 c 1.086526 4.828921 0.000000<br>36 c -0.126870 4.135490 0.000000	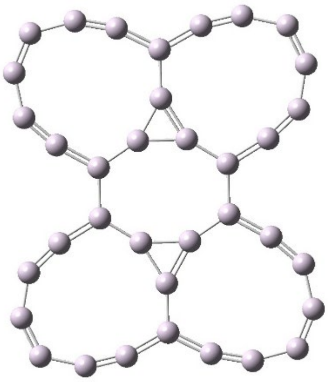
9.	$^{S1}C_{72\text{-sheet-1}}$	

	1 c 0.202843 -10.810484 0.000000<br>2 c -1.016967 -11.490519 0.000000<br>3 c 1.449661 -11.439622 0.000000<br>4 c -2.221050 -11.764905 0.000000<br>5 c 2.664711 -11.661204 0.000000<br>6 c -3.546109 -11.489451 0.000000<br>7 c 3.978041 -11.334765 0.000000<br>8 c -4.069225 -10.353530 0.000000<br>9 c 4.454959 -10.178473 0.000000<br>10 c -3.861856 -9.032828 0.000000<br>11 c 4.198087 -8.866777 0.000000<br>12 c -2.953975 -8.186473 0.000000<br>13 c 3.253540 -8.061359 0.000000<br>14 c -1.767628 -7.459798 0.000000<br>15 c 2.038304 -7.385008 0.000000<br>16 c -0.570304 -8.178307 0.000000<br>17 c 0.870265 -8.150356 0.000000<br>18 c 0.173522 -9.370606 0.000000<br>19 c 0.069436 -4.035817 0.000000<br>20 c -0.623966 -5.255225 0.000000<br>21 c 0.810556 -5.227139 0.000000<br>22 c -1.789190 -5.985794 0.000000<br>23 c 2.002701 -5.911675 0.000000<br>24 c -2.952732 -5.193913 0.000000<br>25 c 3.136468 -5.077314 0.000000	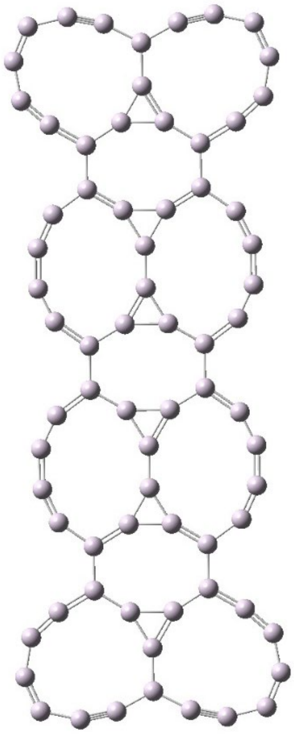

	26 c -3.500166 -4.087131 0.000000<br>27 c 3.647018 -3.953333 0.000000<br>28 c -3.538047 -2.741543 0.000000<br>29 c 3.633878 -2.606724 0.000000<br>30 c -3.021385 -1.620177 0.000000<br>31 c 3.074232 -1.506511 0.000000<br>32 c -1.901528 -0.770319 0.000000<br>33 c 1.921734 -0.700571 0.000000<br>34 c -0.698234 -1.446995 0.000000<br>35 c 0.744510 -1.420782 0.000000<br>36 c 0.044231 -2.636483 0.000000<br>37 c -0.052527 2.635802 0.000000<br>38 c -0.751116 1.419520 0.000000<br>39 c 0.691589 1.446873 0.000000<br>40 c -1.928475 0.699630 0.000000<br>41 c 1.894534 0.769409 0.000000<br>42 c -3.079194 1.507927 0.000000<br>43 c 3.016148 1.616638 0.000000<br>44 c -3.636482 2.609370 0.000000<br>45 c 3.533807 2.737458 0.000000<br>46 c -3.649159 3.955827 0.000000<br>47 c 3.496425 4.083082 0.000000<br>48 c -3.143267 5.081858 0.000000<br>49 c 2.945450 5.188000 0.000000<br>50 c -2.008996 5.915957 0.000000	

	51 c 1.782898 5.980772<br>0.000000<br>52 c -0.818234 5.228612<br>0.000000<br>53 c 0.616216 5.252833<br>0.000000<br>54 c -0.079591 4.035162<br>0.000000<br>55 c -0.171638 9.370148<br>0.000000<br>56 c -0.871617 8.151716<br>0.000000<br>57 c 0.568826 8.175792<br>0.000000<br>58 c -2.041858 7.389882<br>0.000000<br>59 c 1.764361 7.454426<br>0.000000<br>60 c -3.253214 8.073816<br>0.000000<br>61 c 2.953489 8.176014<br>0.000000<br>62 c -4.190663 8.887534<br>0.000000<br>63 c 3.867385 9.015893<br>0.000000<br>64 c -4.445459 10.199586<br>0.000000<br>65 c 4.078488 10.335864<br>0.000000<br>66 c -3.965312 11.354552<br>0.000000<br>67 c 3.559321 11.473713<br>0.000000<br>68 c -2.650852 11.676545<br>0.000000<br>69 c 2.234807 11.751938<br>0.000000<br>70 c -1.437537 11.446032<br>0.000000<br>71 c 1.029356 11.483586<br>0.000000<br>72 c -0.194247 10.810142<br>0.000000	
10.	$^{S1}C_{72\text{-tube-1}}$	

	1 c 0.028166 -1.514165<br>3.239612<br>2 c -1.140877 -0.732387<br>3.539680<br>3 c 1.158981 -0.682346<br>3.546908<br>4 c -2.550080 -0.771959<br>3.912435<br>5 c 2.568073 -0.661318<br>3.922871<br>6 c -3.445914 -1.787175<br>4.034578<br>7 c 3.508085 -1.636489<br>4.044552<br>8 c -3.757360 -2.858803<br>3.469184<br>9 c 3.868986 -2.690628<br>3.476140<br>10 c -3.632478 -3.887794<br>2.641537<br>11 c 3.791183 -3.726003<br>2.650537<br>12 c -2.898166 -4.086322<br>1.643786<br>13 c 3.067451 -3.955978<br>1.651780<br>14 c -1.791560 -4.026480<br>0.843415<br>15 c 1.961680 -3.945344<br>0.848681<br>16 c -0.615503 -3.583134<br>1.551002<br>17 c 0.764622 -3.555062<br>1.552450<br>18 c 0.056682 -2.733697<br>2.459106<br>19 c 0.076726 -3.403234 -<br>2.463306<br>20 c -0.628220 -3.866114 -<br>1.334413<br>21 c 0.798542 -3.835002 -<br>1.332498<br>22 c -1.801203 -4.036239 -<br>0.609323<br>23 c 1.975606 -3.954411 -<br>0.604017<br>24 c -2.994926 -3.873435 -<br>1.350297<br>25 c 3.163800 -3.741957 -<br>1.341567	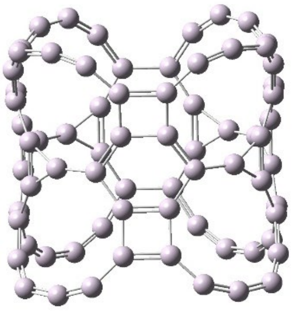

	26 c -3.542666 -3.324553 - 2.306685	
	27 c 3.688333 -3.168941 - 2.296809	
	28 c -3.624732 -2.450721 - 3.339083	
	29 c 3.736071 -2.293718 - 3.330039	
	30 c -2.995167 -1.556589 - 3.909874	
	31 c 3.068733 -1.427321 - 3.900844	
	32 c -1.875477 -0.797509 - 4.289007	
	33 c 1.919335 -0.716474 - 4.283625	
	34 c -0.682612 -1.489769 - 4.164609	
	35 c 0.756715 -1.459059 - 4.162425	
	36 c 0.059002 -2.532778 - 3.574270	
	37 c -0.048463 2.494792 - 3.597302	
	38 c -0.744446 1.415433 - 4.177752	
	39 c 0.694920 1.446498 - 4.175298	
	40 c -1.906723 0.671987 - 4.295683	
	41 c 1.888092 0.753058 - 4.290123	
	42 c -3.058008 1.386293 - 3.924118	
	43 c 3.006293 1.515419 - 3.913757	
	44 c -3.724899 2.258015 - 3.361287	
	45 c 3.635723 2.414682 - 3.350824	
	46 c -3.681656 3.145399 - 2.338226	
	47 c 3.550317 3.297514 - 2.326425	
	48 c -3.157319 3.725734 - 1.387401	
	49 c 3.001313 3.857549 - 1.377251	
	50 c -1.972472 3.947846 - 0.647979	

	51 c 1.804760 4.027582 -<br>0.642048<br>52 c -0.792812 3.820681 -<br>1.371266<br>53 c 0.634069 3.850347 -<br>1.369051<br>54 c -0.068365 3.376447 -<br>2.495246<br>55 c -0.061943 2.760902<br>2.434346<br>56 c -0.769850 3.570513<br>1.517207<br>57 c 0.610233 3.601536<br>1.518515<br>58 c -1.963539 3.954450<br>0.804875<br>59 c 1.790132 4.034254<br>0.810655<br>60 c -3.072361 3.974970<br>1.604237<br>61 c 2.893101 4.102026<br>1.615082<br>62 c -3.797611 3.757020<br>2.604662<br>63 c 3.624604 3.913563<br>2.617003<br>64 c -3.877927 2.731085<br>3.441872<br>65 c 3.746139 2.892006<br>3.454704<br>66 c -3.521056 1.680021<br>4.018914<br>67 c 3.431380 1.828883<br>4.034022<br>68 c -2.582113 0.702883<br>3.906068<br>69 c 2.534924 0.812996<br>3.920258<br>70 c -1.172712 0.720063<br>3.534190<br>71 c 1.126966 0.770687<br>3.541652<br>72 c -0.038523 1.549774<br>3.227212	
11.	$^{S1}C_{72\text{-sheet-2}}$	

	1 c 10.710064 0.000000 0.044499<br>2 c 11.930571 -0.706378 0.070806<br>3 c 11.930571 0.706378 0.070806<br>4 c 12.458754 -2.004545 0.085895<br>5 c 12.458754 2.004545 0.085895<br>6 c 11.597117 -2.956500 0.070013<br>7 c 11.597117 2.956500 0.070013<br>8 c 10.642200 -3.868670 0.054745<br>9 c 10.642200 3.868670 0.054745<br>10 c 9.381025 -3.488230 0.031426<br>11 c 9.381025 3.488230 0.031426<br>12 c 8.168513 -3.099622 0.011532<br>13 c 8.168513 3.099622 0.011532<br>14 c 7.416517 -1.935697 - 0.002831<br>15 c 7.416517 1.935697 - 0.002831<br>16 c 8.109971 -0.704109 0.003562<br>17 c 8.109971 0.704109 0.003562<br>18 c 9.321174 0.000000 0.020548<br>19 c 4.041408 0.000000 - 0.041048<br>20 c 5.251837 -0.714085 - 0.030574<br>21 c 5.251837 0.714085 - 0.030574<br>22 c 5.954220 -1.911375 - 0.020934<br>23 c 5.954220 1.911375 - 0.020934<br>24 c 5.125183 -3.043555 - 0.026536<br>25 c 5.125183 3.043555 - 0.026536	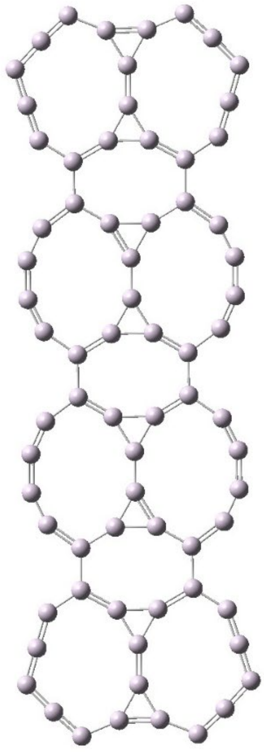

	26 c 4.015993 -3.585414 -0.033685<br>27 c 4.015993 3.585414 -0.033685<br>28 c 2.671497 -3.577107 -0.042533<br>29 c 2.671497 3.577107 -0.042533<br>30 c 1.555011 -3.049630 -0.048754<br>31 c 1.555011 3.049630 -0.048754<br>32 c 0.734894 -1.909181 -0.053861<br>33 c 0.734894 1.909181 -0.053861<br>34 c 1.437613 -0.719837 -0.055119<br>35 c 1.437613 0.719837 -0.055119<br>36 c 2.643234 0.000000 -0.050301<br>37 c -2.643234 0.000000 -0.050301<br>38 c -1.437613 -0.719837 -0.055119<br>39 c -1.437613 0.719837 -0.055119<br>40 c -0.734894 -1.909181 -0.053861<br>41 c -0.734894 1.909181 -0.053861<br>42 c -1.555011 -3.049630 -0.048754<br>43 c -1.555011 3.049630 -0.048754<br>44 c -2.671497 -3.577107 -0.042533<br>45 c -2.671497 3.577107 -0.042533<br>46 c -4.015993 -3.585414 -0.033685<br>47 c -4.015993 3.585414 -0.033685<br>48 c -5.125183 -3.043555 -0.026536<br>49 c -5.125183 3.043555 -0.026536<br>50 c -5.954220 -1.911375 -0.020934	

	51 c -5.954220 1.911375 -<br>0.020934<br>52 c -5.251837 -0.714085 -<br>0.030574<br>53 c -5.251837 0.714085 -<br>0.030574<br>54 c -4.041408 0.000000 -<br>0.041048<br>55 c -9.321174 0.000000<br>0.020548<br>56 c -8.109971 -0.704109<br>0.003562<br>57 c -8.109971 0.704109<br>0.003562<br>58 c -7.416517 -1.935697 -<br>0.002831<br>59 c -7.416517 1.935697 -<br>0.002831<br>60 c -8.168513 -3.099622<br>0.011532<br>61 c -8.168513 3.099622<br>0.011532<br>62 c -9.381025 -3.488230<br>0.031426<br>63 c -9.381025 3.488230<br>0.031426<br>64 c -10.642200 -3.868670<br>0.054745<br>65 c -10.642200 3.868670<br>0.054745<br>66 c -11.597117 -2.956500<br>0.070013<br>67 c -11.597117 2.956500<br>0.070013<br>68 c -12.458754 -2.004545<br>0.085895<br>69 c -12.458754 2.004545<br>0.085895<br>70 c -11.930571 -0.706378<br>0.070806<br>71 c -11.930571 0.706378<br>0.070806<br>72 c -10.710064 0.000000<br>0.044499	
**12.**	$^{S1}C_{72\text{-tube-2}}$	
	1 c 0.017687 -2.491164 -<br>3.582848<br>2 c -0.701889 -1.415347 -<br>4.134293<br>3 c 0.723059 -1.405975 -<br>4.134008	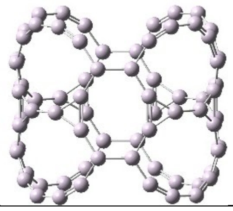

	4 c -1.891310 -0.695463 -<br>4.275618<br>5 c 1.902987 -0.670342 -<br>4.274659<br>6 c -3.029339 -1.449941 -<br>3.946937<br>7 c 3.050475 -1.409729 -<br>3.945226<br>8 c -3.635794 -2.367150 -<br>3.388783<br>9 c 3.669602 -2.319416 -<br>3.388465<br>10 c -3.602908 -3.300720 -<br>2.412263<br>11 c 3.647619 -3.252160 -<br>2.410904<br>12 c -3.029296 -3.892967 -<br>1.496820<br>13 c 3.082759 -3.853029 -<br>1.495638<br>14 c -1.869203 -4.182526 -<br>0.752110<br>15 c 1.926060 -4.156986 -<br>0.751221<br>16 c -0.685421 -4.018956 -<br>1.475444<br>17 c 0.740534 -4.009337 -<br>1.475105<br>18 c 0.024190 -3.481282 -<br>2.564793<br>19 c 0.023322 -3.543491<br>2.527204<br>20 c -0.686110 -4.056049<br>1.425589<br>21 c 0.740389 -4.046303<br>1.425917<br>22 c -1.869719 -4.201475<br>0.698076<br>23 c 1.926169 -4.175531<br>0.698952<br>24 c -3.031742 -3.932743<br>1.447911<br>25 c 3.084281 -3.890811<br>1.449053<br>26 c -3.604323 -3.356112<br>2.373618<br>27 c 3.648107 -3.306012<br>2.375000<br>28 c -3.642379 -2.442525<br>3.368557	

	29 c 3.673773 -2.392170 3.370064	
	30 c -3.030406 -1.534060 3.935345	
	31 c 3.048227 -1.493725 3.937928	
	32 c -1.893481 -0.782594 4.271804	
	33 c 1.901210 -0.757422 4.273559	
	34 c -0.703563 -1.502031 4.127163	
	35 c 0.721052 -1.492673 4.128014	
	36 c 0.016274 -2.571029 3.562820	
	37 c -0.017687 2.491164 3.582848	
	38 c -0.723059 1.405975 4.134008	
	39 c 0.701889 1.415347 4.134293	
	40 c -1.902987 0.670342 4.274659	
	41 c 1.891310 0.695463 4.275618	
	42 c -3.050475 1.409729 3.945226	
	43 c 3.029339 1.449941 3.946937	
	44 c -3.669602 2.319416 3.388465	
	45 c 3.635794 2.367150 3.388783	
	46 c -3.647619 3.252160 2.410904	
	47 c 3.602908 3.300720 2.412263	
	48 c -3.082759 3.853029 1.495638	
	49 c 3.029296 3.892967 1.496820	
	50 c -1.926060 4.156986 0.751221	
	51 c 1.869203 4.182526 0.752110	
	52 c -0.740534 4.009337 1.475105	
	53 c 0.685421 4.018956 1.475444	

	54 c	-0.024190	3.481282	2.564793	
	55 c	-0.023322	3.543491	- 2.527204	
	56 c	-0.740389	4.046303	- 1.425917	
	57 c	0.686110	4.056049	- 1.425589	
	58 c	-1.926169	4.175531	- 0.698952	
	59 c	1.869719	4.201475	- 0.698076	
	60 c	-3.084281	3.890811	- 1.449053	
	61 c	3.031742	3.932743	- 1.447911	
	62 c	-3.648107	3.306012	- 2.375000	
	63 c	3.604323	3.356112	- 2.373618	
	64 c	-3.673773	2.392170	- 3.370064	
	65 c	3.642379	2.442525	- 3.368557	
	66 c	-3.048227	1.493725	- 3.937928	
	67 c	3.030406	1.534060	- 3.935345	
	68 c	-1.901210	0.757422	- 4.273559	
	69 c	1.893481	0.782594	- 4.271804	
	70 c	-0.721052	1.492673	- 4.128014	
	71 c	0.703563	1.502031	- 4.127163	
	72 c	-0.016274	2.571029	- 3.562820	
13.	$^{S2}C_{18}$				
	1 c	-0.721595	0.000000	-0.729585	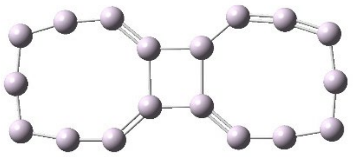
	2 c	0.721595	0.000000	-0.729585	
	3 c	-1.731979	0.000000	-1.605633	
	4 c	1.731979	0.000000	-1.605633	
	5 c	-3.014192	0.000000	-1.602289	
	6 c	3.014192	0.000000	-1.602289	
	7 c	-4.325377	0.000000	-1.379802	
	8 c	4.325377	0.000000	-1.379802	
	9 c	-4.443712	0.000000	-0.082012	
	10 c	4.443712	0.000000	-0.082012	
	11 c	-4.291848	0.000000	1.229492	
	12 c	4.291848	0.000000	1.229492	

	13 c -3.050804 0.000000 1.607466<br>14 c 3.050804 0.000000 1.607466<br>15 c -1.752706 0.000000 1.699829<br>16 c 1.752706 0.000000 1.699829<br>17 c -0.735091 0.000000 0.862533<br>18 c 0.735091 0.000000 0.862533	
**14.**	$^{S2}C_{18}$ (TS1)	
	1 c 0.481533 -0.954170 0.000000<br>2 c -0.781672 -1.402172 0.000000<br>3 c 1.607228 -1.703532 0.000000<br>4 c -2.020011 -1.679490 0.000000<br>5 c 2.864244 -1.837111 0.000000<br>6 c -3.279589 -1.376456 0.000000<br>7 c 4.199496 -1.783781 0.000000<br>8 c -4.428811 -0.782373 0.000000<br>9 c 4.542358 -0.522936 0.000000<br>10 c -4.481608 0.516007 0.000000<br>11 c 4.495241 0.782844 0.000000<br>12 c -4.135133 1.768432 0.000000<br>13 c 3.207029 1.138273 0.000000<br>14 c -2.880932 2.085693 0.000000<br>15 c 1.952534 1.293512 0.000000<br>16 c -1.585450 2.094338 0.000000<br>17 c 0.685967 0.819823 0.000000<br>18 c -0.442423 1.543100 0.000000	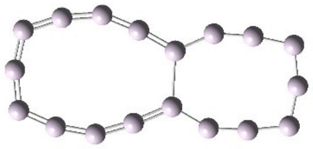
**15.**	$^{S2}C_{18}^{prim}$	
	1 c 0.482352 -0.837350 -0.137773<br>2 c -0.748480 -1.336390 0.129027<br>3 c 1.550066 -1.658564 -0.471933<br>4 c -1.955870 -1.653614 0.363952<br>5 c 2.803811 -1.772843 -0.452398<br>6 c -3.215505 -1.352473 0.396551<br>7 c 4.147212 -1.729068 -0.364315<br>8 c -4.355827 -0.740563 0.343083<br>9 c 4.459375 -0.511228 -0.003572<br>10 c -4.426401 0.510065 -0.000348<br>11 c 4.431504 0.745291 0.358769<br>12 c -4.073184 1.711739 -0.343346	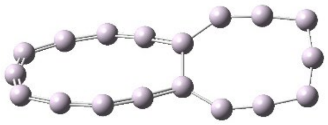

	13 c 3.132813 1.090723<br>0.450370<br>14 c -2.823171 2.047758 -<br>0.395511<br>15 c 1.885547 1.261289<br>0.473454<br>16 c -1.528278 2.053518 -<br>0.361436<br>17 c 0.659359 0.702783<br>0.141149<br>18 c -0.425324 1.468929 -<br>0.125722	
**16.**	$^{S2}C_{18}$ (TS2)	
	1 c 0.642158 -0.815216<br>0.000000<br>2 c -0.795534 -0.763911<br>0.000000<br>3 c 1.523138 -1.831355<br>0.000000<br>4 c -1.923110 -1.433900<br>0.000000<br>5 c 2.802117 -1.931722<br>0.000000<br>6 c -3.204408 -1.265062<br>0.000000<br>7 c 4.123088 -1.819948<br>0.000000<br>8 c -4.452341 -0.850067<br>0.000000<br>9 c 4.407989 -0.545664<br>0.000000<br>10 c -4.424936 0.456913<br>0.000000<br>11 c 4.419796 0.770538<br>0.000000<br>12 c -4.114897 1.732987<br>0.000000<br>13 c 3.200038 1.244639<br>0.000000<br>14 c -2.837752 1.961408<br>0.000000<br>15 c 1.927049 1.472653<br>0.000000<br>16 c -1.536906 1.899682<br>0.000000<br>17 c 0.832675 0.711244<br>0.000000<br>18 c -0.588164 1.006782<br>0.000000	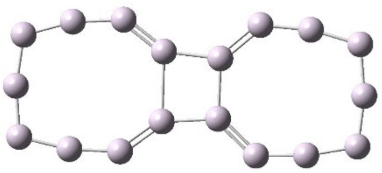
**17.**	$^{S2}C_{36\text{-ribbon-1}}$	

	1 c -5.982701 0.057665 0.000000<br>2 c -5.017452 1.076658 0.000000<br>3 c -7.331697 -0.143554 0.000000<br>4 c -4.806914 2.461461 0.000000<br>5 c -8.072773 -1.179468 0.000000<br>6 c -3.983533 3.381415 0.000000<br>7 c -8.709872 -2.352391 0.000000<br>8 c -2.690708 3.792558 0.000000<br>9 c -7.830993 -3.320672 0.000000<br>10 c -1.501810 3.461354 0.000000<br>11 c -6.745092 -4.046595 0.000000<br>12 c -0.677299 2.293842 0.000000<br>13 c -5.678111 -3.242283 0.000000<br>14 c -1.514203 1.140709 0.000000<br>15 c -4.765819 -2.355075 0.000000<br>16 c -2.539652 0.466719 0.000000<br>17 c -4.765638 -0.990626 0.000000<br>18 c -3.894518 0.111372 0.000000<br>19 c 5.032210 1.005467 0.000000<br>20 c 5.982906 -0.027108 0.000000<br>21 c 4.841482 2.393167 0.000000<br>22 c 7.328915 -0.247427 0.000000<br>23 c 4.030907 3.324423 0.000000<br>24 c 8.055222 -1.293741 0.000000<br>25 c 2.744180 3.754261 0.000000	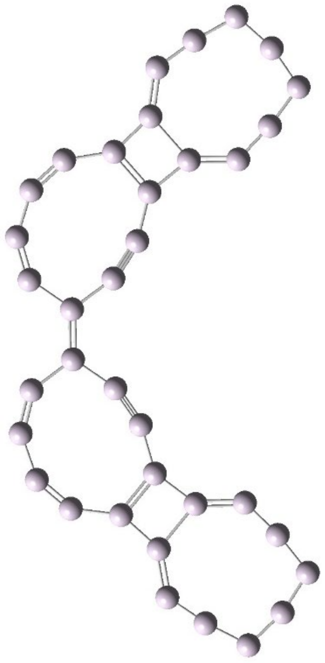

	26 c 8.675658 -2.475556<br>0.000000<br>27 c 1.550843 3.439486<br>0.000000<br>28 c 7.783168 -3.431306<br>0.000000<br>29 c 0.709678 2.283944<br>0.000000<br>30 c 6.687099 -4.141782<br>0.000000<br>31 c 1.530098 1.119036<br>0.000000<br>32 c 5.631608 -3.322429<br>0.000000<br>33 c 2.545997 0.430718<br>0.000000<br>34 c 4.731988 -2.422377<br>0.000000<br>35 c 3.895707 0.056191<br>0.000000<br>36 c 4.751119 -1.058054<br>0.000000	
18.	$^{S2}C_{36\text{-ribbon-1}}$ (TS)	
	1 c 6.361480 0.000000<br>0.389191<br>2 c 5.116854 0.000000<br>1.043993<br>3 c 7.701391 0.000000<br>0.636969<br>4 c 4.493342 0.000000<br>2.295773<br>5 c 8.740215 0.000000 -<br>0.102457<br>6 c 3.369690 0.000000<br>2.831375<br>7 c 9.724073 0.000000 -<br>1.001751<br>8 c 2.038929 0.000000<br>2.961851<br>9 c 9.208609 0.000000 -<br>2.205462<br>10 c 0.975408 0.000000<br>2.275578<br>11 c 8.419501 0.000000 -<br>3.244695<br>12 c 0.698129 0.000000<br>0.846165<br>13 c 7.148436 0.000000 -<br>2.829206<br>14 c 1.786845 0.000000 -<br>0.034707	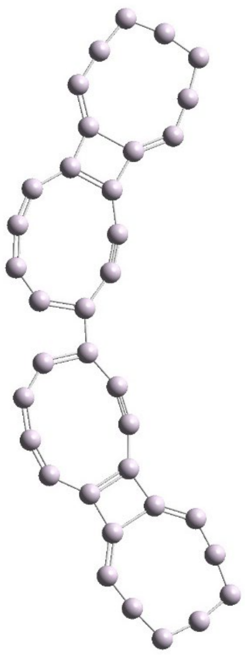

	15 c 5.996880 0.000000 -<br>2.288388<br>16 c 2.979267 0.000000 -<br>0.336089<br>17 c 5.554510 0.000000 -<br>0.997897<br>18 c 4.371291 0.000000 -<br>0.240243<br>19 c -5.116854 0.000000<br>1.043993<br>20 c -6.361480 0.000000<br>0.389191<br>21 c -4.493342 0.000000<br>2.295773<br>22 c -7.701391 0.000000<br>0.636969<br>23 c -3.369690 0.000000<br>2.831375<br>24 c -8.740215 0.000000 -<br>0.102457<br>25 c -2.038929 0.000000<br>2.961851<br>26 c -9.724073 0.000000 -<br>1.001751<br>27 c -0.975408 0.000000<br>2.275578<br>28 c -9.208609 0.000000 -<br>2.205462<br>29 c -0.698129 0.000000<br>0.846165<br>30 c -8.419501 0.000000 -<br>3.244695<br>31 c -1.786845 0.000000 -<br>0.034707<br>32 c -7.148436 0.000000 -<br>2.829206<br>33 c -2.979267 0.000000 -<br>0.336089<br>34 c -5.996880 0.000000 -<br>2.288388<br>35 c -4.371291 0.000000 -<br>0.240243<br>36 c -5.554510 0.000000 -<br>0.997897	
19.	$^{S2}C_{36\text{-ribbon-2}}$	

	1 c -6.346158 0.499388 0.000000<br>2 c -5.048172 1.051790 0.000000<br>3 c -7.657577 0.863348 0.000000<br>4 c -4.333071 2.244795 0.000000<br>5 c -8.758334 0.216921 0.000000<br>6 c -3.132125 2.628220 0.000000<br>7 c -9.814167 -0.594094 0.000000<br>8 c -1.825710 2.786874 0.000000<br>9 c -9.403279 -1.838432 0.000000<br>10 c -0.751720 2.031831 0.000000<br>11 c -8.707061 -2.941256 0.000000<br>12 c -0.701584 0.536507 0.000000<br>13 c -7.404823 -2.638580 0.000000<br>14 c -1.829734 -0.268679 0.000000<br>15 c -6.211060 -2.196839 0.000000<br>16 c -3.040677 -0.500398 0.000000<br>17 c -5.662683 -0.948643 0.000000<br>18 c -4.415291 -0.298177 0.000000<br>19 c 5.061586 0.984285 0.000000<br>20 c 6.352038 0.414572 0.000000<br>21 c 4.362909 2.187118 0.000000<br>22 c 7.668250 0.760881 0.000000<br>23 c 3.166760 2.585345 0.000000<br>24 c 8.760352 0.099945 0.000000<br>25 c 1.862644 2.763045 0.000000	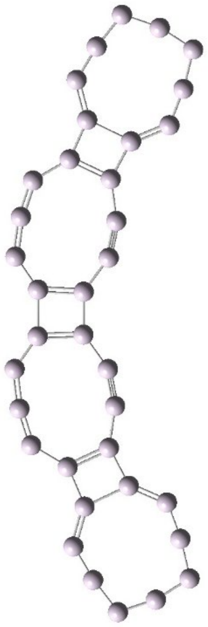

	26 c 9.805366 -0.724940 0.000000<br>27 c 0.779152 2.021614 0.000000<br>28 c 9.377946 -1.963757 0.000000<br>29 c 0.708842 0.527122 0.000000<br>30 c 8.667191 -3.057222 0.000000<br>31 c 1.825934 -0.293295 0.000000<br>32 c 7.369025 -2.737132 0.000000<br>33 c 3.033685 -0.541136 0.000000<br>34 c 6.181278 -2.279609 0.000000<br>35 c 4.410850 -0.357212 0.000000<br>36 c 5.649417 -1.024200 0.000000	
**20.**	$^{S2}C_{72}$-ribbon	
	1 c 12.076686 0.601763 - 6.276475<br>2 c 11.900566 0.439032 - 4.881238<br>3 c 13.050442 0.737776 - 7.220348<br>4 c 12.587990 0.242934 - 3.691029<br>5 c 13.057793 0.846400 - 8.491569<br>6 c 12.243134 0.261947 - 2.462811<br>7 c 12.902539 0.940524 - 9.811315<br>8 c 11.812571 0.122062 - 1.230061<br>9 c 11.621966 0.915933 - 10.096897<br>10 c 10.588921 0.293576 - 0.695893<br>11 c 10.321017 0.851732 - 10.067734<br>12 c 9.282617 0.370977 - 1.416320<br>13 c 9.923473 0.750624 - 8.790198<br>14 c 9.150301 0.386448 - 2.790361	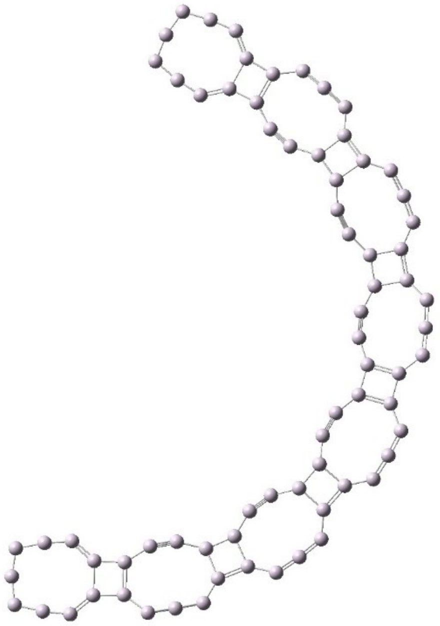

	15 c 9.702436 0.639594 -<br>7.545340<br>16 c 9.531499 0.409517 -<br>3.963935<br>17 c 10.494096 0.581828 -<br>6.430485<br>18 c 10.407904 0.460778 -<br>5.035618<br>19 c 6.869074 -0.738760<br>3.657112<br>20 c 5.656494 -0.879326<br>4.464773<br>21 c 8.196145 -0.452190<br>3.725902<br>22 c 5.165870 -0.767992<br>5.706404<br>23 c 9.125999 -0.206439<br>2.878225<br>24 c 3.962494 -0.714175<br>6.193785<br>25 c 9.946703 0.074039<br>1.910064<br>26 c 2.741643 -0.643891<br>6.597443<br>27 c 9.843712 0.233683<br>0.591266<br>28 c 1.555209 -0.613211<br>5.926288<br>29 c 8.554559 0.270659 -<br>0.204408<br>30 c 1.352306 -0.542053<br>4.431300<br>31 c 7.340652 -0.089833<br>0.318902<br>32 c 2.352770 -0.665815<br>3.532231<br>33 c 6.543943 -0.503713<br>1.177362<br>34 c 3.509380 -0.869105<br>3.094910<br>35 c 6.064145 -0.852964<br>2.412652<br>36 c 4.850700 -1.013140<br>3.165289<br>37 c -4.199160 -0.199412<br>5.804150<br>38 c -5.590903 -0.228977<br>5.342537<br>39 c -3.394112 -0.441904<br>6.852476	

	40 c -6.853841 -0.529221<br>5.735438	
	41 c -2.122454 -0.590444<br>7.019159	
	42 c -7.986630 -0.595827<br>5.124943	
	43 c -0.834599 -0.699767<br>7.078679	
	44 c -9.053674 -0.703228<br>4.397918	
	45 c 0.125910 -0.682659<br>6.121698	
	46 c -9.313134 -0.597348<br>3.088524	
	47 c -0.079029 -0.544820<br>4.617529	
	48 c -8.280551 -0.516652<br>1.968947	
	49 c -1.265982 -0.264699<br>4.045741	
	50 c -6.960477 -0.267964<br>2.196320	
	51 c -2.487510 0.009174<br>3.960695	
	52 c -5.936201 -0.020884<br>2.863158	
	53 c -3.759953 0.144689<br>4.392044	
	54 c -5.139616 0.103626<br>3.959907	
	55 c -12.786877 0.355816 -<br>1.473621	
	56 c -13.357566 0.420118 -<br>2.768419	
	57 c -13.117889 0.337130 -<br>0.126149	
	58 c -14.563741 0.406050 -<br>3.404705	
	59 c -12.421509 0.064024<br>0.914112	
	60 c -14.941938 0.492645 -<br>4.619394	
	61 c -11.685673 -0.053526<br>1.994489	
	62 c -15.177801 0.604604 -<br>5.926551	
	63 c -10.366758 -0.367559<br>2.086855	
	64 c -14.033308 0.699222 -<br>6.562083	

	65 c -9.323066 -0.332816<br>1.020083<br>66 c -12.778461 0.787090 -<br>6.900986<br>67 c -9.569977 -0.043042 -<br>0.304626<br>68 c -12.028908 0.697209 -<br>5.789986<br>69 c -10.254116 0.200190 -<br>1.303590<br>70 c -11.457292 0.619645 -<br>4.660519<br>71 c -11.400538 0.371515 -<br>2.059381<br>72 c -11.890415 0.488785 -<br>3.367267		
**21.**	S2C_{36}-sheet		
	1 c -0.698665 0.000000 -<br>3.121878<br>2 c 0.698665 0.000000 -<br>3.121878<br>3 c -1.688698 0.000000 -<br>4.089939<br>4 c 1.688698 0.000000 -<br>4.089939<br>5 c -2.848464 0.000000 -<br>4.517581<br>6 c 2.848464 0.000000 -<br>4.517581<br>7 c -4.221104 0.000000 -<br>4.418998<br>8 c 4.221104 0.000000 -<br>4.418998<br>9 c -4.563099 0.000000 -<br>3.207836<br>10 c 4.563099 0.000000 -<br>3.207836<br>11 c -4.191843 0.000000 -<br>1.917505<br>12 c 4.191843 0.000000 -<br>1.917505<br>13 c -3.095871 0.000000 -<br>1.332320<br>14 c 3.095871 0.000000 -<br>1.332320<br>15 c -1.809077 0.000000 -<br>0.742060<br>16 c 1.809077 0.000000 -<br>0.742060<br>17 c -0.738111 0.000000 -<br>1.613297	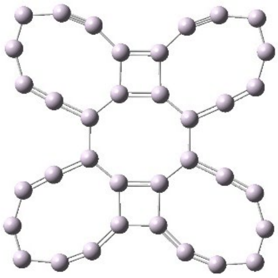	

	18 c 0.738111 0.000000 -<br>1.613297<br>19 c -0.737666 0.000000<br>1.613024<br>20 c 0.737666 0.000000<br>1.613024<br>21 c -1.809138 0.000000<br>0.742496<br>22 c 1.809138 0.000000<br>0.742496<br>23 c -3.095668 0.000000<br>1.334244<br>24 c 3.095668 0.000000<br>1.334244<br>25 c -4.191289 0.000000<br>1.919272<br>26 c 4.191289 0.000000<br>1.919272<br>27 c -4.571977 0.000000<br>3.207647<br>28 c 4.571977 0.000000<br>3.207647<br>29 c -4.219102 0.000000<br>4.414525<br>30 c 4.219102 0.000000<br>4.414525<br>31 c -2.848069 0.000000<br>4.521681<br>32 c 2.848069 0.000000<br>4.521681<br>33 c -1.691040 0.000000<br>4.086763<br>34 c 1.691040 0.000000<br>4.086763<br>35 c -0.698620 0.000000<br>3.121763<br>36 c 0.698620 0.000000<br>3.121763	
22.	$^{S2}C_{72\text{-sheet-1}}$	

	1 c -0.789571 -7.278273 - 0.700502	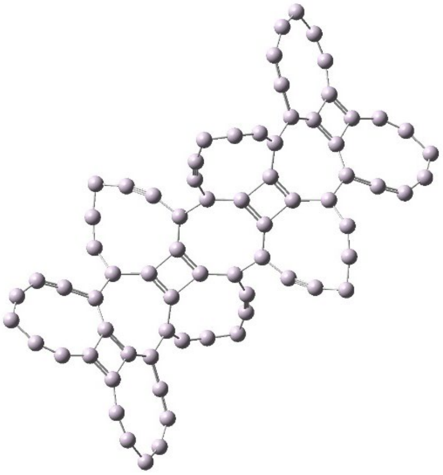
	2 c -0.789571 -7.278273 0.700502	
	3 c -1.187509 -8.142904 - 1.697499	
	4 c -1.187509 -8.142904 1.697499	
	5 c -1.159392 -8.572190 - 2.860910	
	6 c -1.159392 -8.572190 2.860910	
	7 c -0.810025 -8.620851 - 4.182634	
	8 c -0.810025 -8.620851 4.182634	
	9 c 0.007903 -7.694400 - 4.443219	
	10 c 0.007903 -7.694400 4.443219	
	11 c 0.663262 -6.592300 - 4.066475	
	12 c 0.663262 -6.592300 4.066475	
	13 c 0.779106 -5.971576 - 2.994035	
	14 c 0.779106 -5.971576 2.994035	
	15 c 0.763944 -5.350157 - 1.727669	
	16 c 0.763944 -5.350157 1.727669	
	17 c 0.050663 -6.006154 - 0.715452	
	18 c 0.050663 -6.006154 0.715452	
	19 c 1.142070 -3.026655 - 0.692007	
	20 c 1.142070 -3.026655 0.692007	
	21 c 1.460414 -4.071339 - 1.681525	
	22 c 1.460414 -4.071339 1.681525	
	23 c 2.298468 -3.801806 - 2.735089	
	24 c 2.298468 -3.801806 2.735089	
	25 c 2.716930 -2.949376 - 3.569282	

	26 c 2.716930 -2.949376 3.569282	
	27 c 2.868227 -1.914241 - 4.427576	
	28 c 2.868227 -1.914241 4.427576	
	29 c 1.979866 -1.155932 - 3.722752	
	30 c 1.979866 -1.155932 3.722752	
	31 c 1.125485 -0.722816 - 2.914832	
	32 c 1.125485 -0.722816 2.914832	
	33 c 0.456068 -0.597772 - 1.717344	
	34 c 0.456068 -0.597772 1.717344	
	35 c 0.695872 -1.564916 - 0.713977	
	36 c 0.695872 -1.564916 0.713977	
	37 c -0.695872 1.564916 - 0.713977	
	38 c -0.695872 1.564916 0.713977	
	39 c -0.456068 0.597772 - 1.717344	
	40 c -0.456068 0.597772 1.717344	
	41 c -1.125485 0.722816 - 2.914832	
	42 c -1.125485 0.722816 2.914832	
	43 c -1.979866 1.155932 - 3.722752	
	44 c -1.979866 1.155932 3.722752	
	45 c -2.868227 1.914241 - 4.427576	
	46 c -2.868227 1.914241 4.427576	
	47 c -2.716930 2.949376 - 3.569282	
	48 c -2.716930 2.949376 3.569282	
	49 c -2.298468 3.801806 - 2.735089	
	50 c -2.298468 3.801806 2.735089	

	51 c -1.460414 4.071339 -<br>1.681525<br>52 c -1.460414 4.071339<br>1.681525<br>53 c -1.142070 3.026655 -<br>0.692007<br>54 c -1.142070 3.026655<br>0.692007<br>55 c -0.050663 6.006154 -<br>0.715452<br>56 c -0.050663 6.006154<br>0.715452<br>57 c -0.763944 5.350157 -<br>1.727669<br>58 c -0.763944 5.350157<br>1.727669<br>59 c -0.779106 5.971576 -<br>2.994035<br>60 c -0.779106 5.971576<br>2.994035<br>61 c -0.663262 6.592300 -<br>4.066475<br>62 c -0.663262 6.592300<br>4.066475<br>63 c -0.007903 7.694400 -<br>4.443219<br>64 c -0.007903 7.694400<br>4.443219<br>65 c 0.810025 8.620851 -<br>4.182634<br>66 c 0.810025 8.620851<br>4.182634<br>67 c 1.159392 8.572190 -<br>2.860910<br>68 c 1.159392 8.572190<br>2.860910<br>69 c 1.187509 8.142904 -<br>1.697499<br>70 c 1.187509 8.142904<br>1.697499<br>71 c 0.789571 7.278273 -<br>0.700502<br>72 c 0.789571 7.278273<br>0.700502	
23.	$^{S2}C_{72}$-sheet-2	

	1 c 0.000000 0.699043 -<br>9.307554<br>2 c 0.000000 -0.699043 -<br>9.307554<br>3 c 0.000000 1.691876 -<br>10.270052<br>4 c 0.000000 -1.691876 -<br>10.270052<br>5 c 0.000000 2.855395 -<br>10.690361<br>6 c 0.000000 -2.855395 -<br>10.690361<br>7 c 0.000000 4.226113 -<br>10.588368<br>8 c 0.000000 -4.226113 -<br>10.588368<br>9 c 0.000000 4.565644 -<br>9.375673<br>10 c 0.000000 -4.565644 -<br>9.375673<br>11 c 0.000000 4.199870 -<br>8.084859<br>12 c 0.000000 -4.199870 -<br>8.084859<br>13 c 0.000000 3.102829 -<br>7.500846<br>14 c 0.000000 -3.102829 -<br>7.500846<br>15 c 0.000000 1.811078 -<br>6.921272<br>16 c 0.000000 -1.811078 -<br>6.921272<br>17 c 0.000000 0.735146 -<br>7.795569<br>18 c 0.000000 -0.735146 -<br>7.795569<br>19 c 0.000000 0.765721 -<br>4.566337<br>20 c 0.000000 -0.765721 -<br>4.566337<br>21 c 0.000000 1.824254 -<br>5.451486<br>22 c 0.000000 -1.824254 -<br>5.451486<br>23 c 0.000000 3.086152 -<br>4.795464<br>24 c 0.000000 -3.086152 -<br>4.795464<br>25 c 0.000000 3.752176 -<br>3.764366	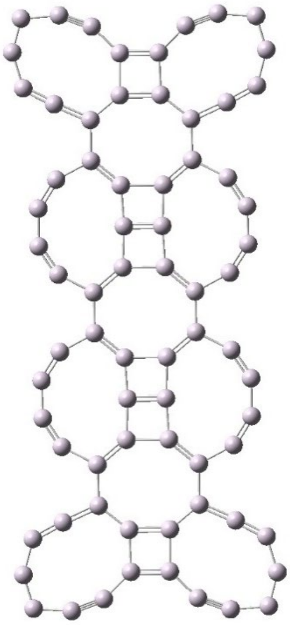

	26 c 0.000000 -3.752176 -<br>3.764366<br>27 c 0.000000 3.766979 -<br>2.407400<br>28 c 0.000000 -3.766979 -<br>2.407400<br>29 c 0.000000 3.083852 -<br>1.387581<br>30 c 0.000000 -3.083852 -<br>1.387581<br>31 c 0.000000 1.823939 -<br>0.729571<br>32 c 0.000000 -1.823939 -<br>0.729571<br>33 c 0.000000 0.761685 -<br>1.615734<br>34 c 0.000000 -0.761685 -<br>1.615734<br>35 c 0.000000 0.690517 -<br>3.094694<br>36 c 0.000000 -0.690517 -<br>3.094694<br>37 c 0.000000 0.690517<br>3.094694<br>38 c 0.000000 -0.690517<br>3.094694<br>39 c 0.000000 0.761685<br>1.615734<br>40 c 0.000000 -0.761685<br>1.615734<br>41 c 0.000000 1.823939<br>0.729571<br>42 c 0.000000 -1.823939<br>0.729571<br>43 c 0.000000 3.083852<br>1.387581<br>44 c 0.000000 -3.083852<br>1.387581<br>45 c 0.000000 3.766979<br>2.407400<br>46 c 0.000000 -3.766979<br>2.407400<br>47 c 0.000000 3.752176<br>3.764366<br>48 c 0.000000 -3.752176<br>3.764366<br>49 c 0.000000 3.086152<br>4.795464<br>50 c 0.000000 -3.086152<br>4.795464	

	51 c 0.000000 1.824254 5.451486<br>52 c 0.000000 -1.824254 5.451486<br>53 c 0.000000 0.765721 4.566337<br>54 c 0.000000 -0.765721 4.566337<br>55 c 0.000000 0.735146 7.795569<br>56 c 0.000000 -0.735146 7.795569<br>57 c 0.000000 1.811078 6.921272<br>58 c 0.000000 -1.811078 6.921272<br>59 c 0.000000 3.102829 7.500846<br>60 c 0.000000 -3.102829 7.500846<br>61 c 0.000000 4.199870 8.084859<br>62 c 0.000000 -4.199870 8.084859<br>63 c 0.000000 4.565644 9.375673<br>64 c 0.000000 -4.565644 9.375673<br>65 c 0.000000 4.226113 10.588368<br>66 c 0.000000 -4.226113 10.588368<br>67 c 0.000000 2.855395 10.690361<br>68 c 0.000000 -2.855395 10.690361<br>69 c 0.000000 1.691876 10.270052<br>70 c 0.000000 -1.691876 10.270052<br>71 c 0.000000 0.699043 9.307554<br>72 c 0.000000 -0.699043 9.307554	
24.	$^{S2}C_{72}$-tube	

	1 c -2.192093 -0.752114<br>2.800452<br>2 c -2.192093 0.752114<br>2.800452<br>3 c -1.419152 -1.634716<br>3.393807<br>4 c -1.419152 1.634716<br>3.393807<br>5 c -0.744036 -2.678449<br>3.896671<br>6 c -0.744036 2.678449<br>3.896671<br>7 c -1.641784 -3.777176<br>4.039003<br>8 c -1.641784 3.777176<br>4.039003<br>9 c -2.710620 -4.227369<br>3.583855<br>10 c -2.710620 4.227369<br>3.583855<br>11 c -3.378127 -3.795978<br>2.468001<br>12 c -3.378127 3.795978<br>2.468001<br>13 c -3.612178 -3.016567<br>1.543712<br>14 c -3.612178 3.016567<br>1.543712<br>15 c -3.484324 -1.836943<br>0.762764<br>16 c -3.484324 1.836943<br>0.762764<br>17 c -2.983557 -0.697726<br>1.521323<br>18 c -2.983557 0.697726<br>1.521323<br>19 c -3.200611 -0.752130 -<br>1.505166<br>20 c -3.200611 0.752130 -<br>1.505166<br>21 c -3.542117 -1.867303 -<br>0.628290<br>22 c -3.542117 1.867303 -<br>0.628290<br>23 c -3.595504 -3.077547 -<br>1.372128<br>24 c -3.595504 3.077547 -<br>1.372128<br>25 c -3.157638 -3.667925 -<br>2.362640	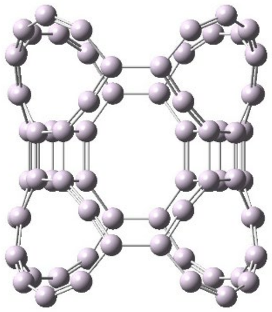

	26 c -3.157638 3.667925 -<br>2.362640<br>27 c -2.324400 -3.669098 -<br>3.433686<br>28 c -2.324400 3.669098 -<br>3.433686<br>29 c -1.440868 -3.085296 -<br>4.066647<br>30 c -1.440868 3.085296 -<br>4.066647<br>31 c -0.696079 -1.880658 -<br>4.183005<br>32 c -0.696079 1.880658 -<br>4.183005<br>33 c -1.498080 -0.743473 -<br>3.725041<br>34 c -1.498080 0.743473 -<br>3.725041<br>35 c -2.513200 -0.741736 -<br>2.732984<br>36 c -2.513200 0.741736 -<br>2.732984<br>37 c 2.513200 -0.741736 -<br>2.732984<br>38 c 2.513200 0.741736 -<br>2.732984<br>39 c 1.498080 -0.743473 -<br>3.725041<br>40 c 1.498080 0.743473 -<br>3.725041<br>41 c 0.696079 -1.880658 -<br>4.183005<br>42 c 0.696079 1.880658 -<br>4.183005<br>43 c 1.440868 -3.085296 -<br>4.066647<br>44 c 1.440868 3.085296 -<br>4.066647<br>45 c 2.324400 -3.669098 -<br>3.433686<br>46 c 2.324400 3.669098 -<br>3.433686<br>47 c 3.157638 -3.667925 -<br>2.362640<br>48 c 3.157638 3.667925 -<br>2.362640<br>49 c 3.595504 -3.077547 -<br>1.372128<br>50 c 3.595504 3.077547 -<br>1.372128	

	51 c 3.542117 -1.867303 -<br>0.628290<br>52 c 3.542117 1.867303 -<br>0.628290<br>53 c 3.200611 -0.752130 -<br>1.505166<br>54 c 3.200611 0.752130 -<br>1.505166<br>55 c 2.983557 -0.697726<br>1.521323<br>56 c 2.983557 0.697726<br>1.521323<br>57 c 3.484324 -1.836943<br>0.762764<br>58 c 3.484324 1.836943<br>0.762764<br>59 c 3.612178 -3.016567<br>1.543712<br>60 c 3.612178 3.016567<br>1.543712<br>61 c 3.378127 -3.795978<br>2.468001<br>62 c 3.378127 3.795978<br>2.468001<br>63 c 2.710620 -4.227369<br>3.583855<br>64 c 2.710620 4.227369<br>3.583855<br>65 c 1.641784 -3.777176<br>4.039003<br>66 c 1.641784 3.777176<br>4.039003<br>67 c 0.744036 -2.678449<br>3.896671<br>68 c 0.744036 2.678449<br>3.896671<br>69 c 1.419152 -1.634716<br>3.393807<br>70 c 1.419152 1.634716<br>3.393807<br>71 c 2.192093 -0.752114<br>2.800452<br>72 c 2.192093 0.752114<br>2.800452	

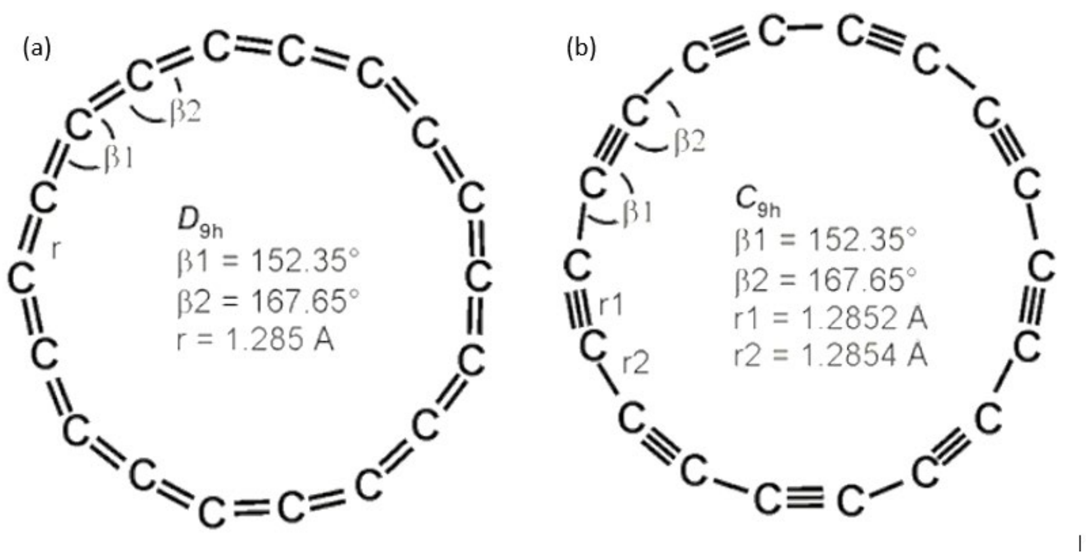

Figure S1. Quantum chemistry calculated[a] structures for cyclo[18]carbon; (a) cumulenic; (b) polyynic.

[a] Gas-phase electronic spectra of C 18 and C 22 rings

Boguslavskiy, A.E.; Ding, H.; Maier, J.P. Gas-phase electronic spectra of C18 and C22 rings. *J. Chem. Phys.* **2005**, *123*, 034305. https://doi.org/10.1063/1.1961564
